# Profiling the barriers to the spreading of news using news headlines

**DOI:** 10.3389/frai.2023.1225213

**Published:** 2023-08-29

**Authors:** Abdul Sittar, Dunja Mladenić, Marko Grobelnik

**Affiliations:** ^1^Information and Communication Technologies, Jožef Stefan International Postgraduate School (IPS), Ljubljana, Slovenia; ^2^Department for Artificial Intelligence - E3, Jozef Stefan Institute, Ljubljana, Slovenia

**Keywords:** news spreading barriers, profiling news spreading barriers, common-sense inferences, sentiment analysis, economic barrier, political barrier, cultural barrier, linguistic barrier

## Abstract

News headlines can be a good data source for detecting the barriers to the spreading of news in news media, which can be useful in many real-world applications. In this study, we utilize semantic knowledge through the inference-based model COMET and the sentiments of news headlines for barrier classification. We consider five barriers, including cultural, economic, political, linguistic, and geographical and different types of news headlines, including health, sports, science, recreation, games, homes, society, shopping, computers, and business. To that end, we collect and label the news headlines automatically for the barriers using the metadata of news publishers. Then, we utilize the extracted common-sense inferences and sentiments as features to detect the barriers to the spreading of news. We compare our approach to the classical text classification methods, deep learning, and transformer-based methods. The results show that (1) the inference-based semantic knowledge provides distinguishable inferences across the 10 categories that can increase the effectiveness and enhance the speed of the classification model; (2) the news of positive sentiments cross the political barrier, whereas the news of negative sentiments cross the cultural, economic, linguistic, and geographical barriers; (3) the proposed approach using inferences-based semantic knowledge and sentiment improves performance compared with using only headlines in barrier classification. The average F1-score for 4 out of 5 barriers has significantly improved as follows: for cultural barriers from 0.41 to 0.47, for economic barriers from 0.39 to 0.55, for political barriers from 0.59 to 0.70 and for geographical barriers from 0.59 to 0.76.

## 1. Introduction

News spreading comes across many barriers due to different reasons including cultural, economic, political, linguistic, or geographical. The term barrier refers to the abstract fences that are in place between different societies, nations, and countries while transferring information. We know that the storylines of the news are anchored to the time, places, or entities; therefore, the coverage of news is hampered by news publisher's preferences (Rospocher et al., 2016; Sittar et al., 2022b). The roots of the existence of the mentioned barriers relate to their influences. The classification of such barriers can be useful in the context of numerous real-world applications, such as trend detection and content recommendations for readers and subscribers (Heydari et al., 2015; Gulla et al., 2017). Thus, it is highly important to classify the barriers to massive news spreading related to different events.

Culture is multifaceted, subsuming behaviors, values, and attitudes that are dominant and unique to a particular group of people. The news media has a strong relationship with many macro-level factors in society, ranging from the economy, governments, the public, and other organizational structures (Ng and Tan, 2021). Within a cultural barrier, media diversity provides different opinions and perspectives across different cultures (d'Haenens et al., 2009). The publishing language of a news media also influences the diffusion of news about global and local events (Wright, 2022), so we can say that there is a language barrier. Similarly, the political alignment of news publishers has a direct relationship with the published content, and it is called as a political barrier by Sittar et al. (2022b). Another contextual variable that has a direct relationship with different types of news is the economic situation (we call it the economic barrier), surrounding the news publisher. Since the economic differences in living styles affect the need, the news is likely to propagate according to the needs of locals.

Another important variable in this context is news sentiments about different events across different locations. Several studies use sentiment from textual data, including social media, and news articles, to forecast financial variables (Barbaglia et al., 2022; Consoli et al., 2022; Kumbure et al., 2022). The sentiment of the news plays an important role in news spreading, as Bustos et al. (2011) found that the price movement on the stock exchange has a direct relationship with the news spreading patterns. Similarly, news about global events has different sentiment polarities across the geographical barrier. Moreo et al. (2012) calls it the popularity measurement of news in a global context. Market behavior is also predictable through sentiments (Godbole et al., 2007; Shah et al., 2018), and sentiments can vary by demographic group, news source, and geographic location (Mehler et al., 2006).

When it comes to news headlines, they reflect the vital information of news articles. It reduces the interpretation time and effort of reading the whole article (Shrawankar and Wankhede, 2016). The first thing in the news article is its headline, which makes the first and foremost impression on the news readers. Plenty of news articles are published every day and spread *via* news and social media (Nassirtoussi et al., 2015; Gabielkov et al., 2016; Gravanis et al., 2019). These headlines have different emotional scores with a negative, positive, or neutral polarity, which directly impacts the readers' actions (Aslam et al., 2020).

Barrier classification with news headlines is a challenging task due to incorporating insufficient information as well as misinformation in the headlines. News coverage in different fields, including sports, health, and computers, has different impact levels. We focus on five different types of barriers, including cultural, political, linguistic, economic, and geographic, as these are important barriers that can influence news spreading (Sittar et al., 2022b). In this study, we assume that common sense-based semantic knowledge and sentiments of news headlines will help to classify barriers to the spreading of news. We are interested in exploring the variations in sentiments across different barriers where news headlines belong to different events. We explore a range of different common-sense descriptions generated by the Natural Language Processing Knowledge Inference Tool (Ismayilzada and Bosselut, 2022). In addition, we present an approach to barrier classification that aims to classify barriers across the news. This approach combines information based on news headlines, their inferences, and their sentiment.

The contributions of this research can be summarized as follows:

1. A novel approach to information barrier annotation based on news meta-data.
2. A dataset for the barrier classification in the news that has been labeled automatically using the metadata and the semantic similarity.
3. An approach to the classification of barriers to the spreading of news based on semantic knowledge, including a wide range of common sense inferences and sentiments of news headlines.

The rest of the study is organized as follows: Section 2 reviews the related work on barrier classification; Section 3 presents the approach; Section 4 describes the benchmark dataset construction; Section 5 discusses the experimental results; Section 8 concludes the study and highlights the theoretical and practical implications of our study.

## 2. Related work

In this section, we present the related work on barriers to the spreading of news and the role of semantic knowledge and sentiments of the news headlines for different tasks.

### 2.1. Barriers to the spreading of news

Effective dissemination is the key to bridging the gap in information spreading. For the scientists and practitioners, it is necessary to participate in explicit, accurate, and unbiased dissemination of their respective areas of expertise to the public (Kelly et al., 2019). The result of communication is not only situation-specific but also inherently culturally bound because it is entrenched in human acts with intentions, interests, and wants as well as larger institutional, social, and cultural systems (Jiang and Tang, 2020). Culture-specific ideology is defined as the values, beliefs, attitudes, or interests expressed in a source text that is associated with a particular culture or source and that may be viewed as undesirable or incompatible with the dominant values, beliefs, attitudes, or interests of another culture or subculture. It defines the strategies adopted by text producers to bridge the divides in global news transmission. According to MCNelly's theory, the more distance an intermediary communicator has to travel before learning about a news occurrence, the less personally invested he is in it and the more he considers its “marketability” to editors or readers (Vuorinen, 1994). It has been said that countries with close distances share culture, and the news reporting on the same events will not differ due to ideology, culture, and geopolitics (Segev, 2015; Ma et al., 2017). Countries that share a common culture are expected to have heavier news flow between them when reporting on similar events (Wu, 2007). There are many quantitative studies that found demographic, psychological, sociocultural, source, system, and content-related aspects (Al-Samarraie et al., 2017).

The role of content is an essential research topic in news spreading. Media economics scholars especially showed their interest in a variety of content forms since content analysis plays a vital role in individual consumer decisions and political and economic interactions (Fico et al., 2008). In content, a frame is a means to highlight certain elements of a seen reality in a communication text, so as to support a specific problem definition, causal interpretation, moral assessment, and/or therapy proposal for the thing being described. There are four places where frames can be found during communication, such as text, recipient, communicator, and culture (Reese, 2007). The inverted pyramid reporting method, where the most significant facts are presented in order of importance, is a key component of news framing. Bias in the news can manifest in a variety of ways, such as “source bias,” “unbalanced presentation of contested themes,” and “frequent usage of packaged formula” (Walter and Ophir, 2019). Scheufele identifies five factors that influence how journalists frame news. These include societal expectations and ideals, organizational demands and restrictions, pressure from interest groups, journalistic practices, and journalists' ideological or political leanings (Obijiofor, 2010). A vast body of literature exists on how the news media frame news events and consequently influence public perception of those events (Lamidi and Olisa, 2016). Existing literature posits that framing is often used intentionally for the purpose of changing the perception of content, and to cater to this, different computational methods have been applied (King et al., 2017; Sheshadri et al., 2021).

### 2.2. Inference-based semantic knowledge

Common-sense transformer (COMET) is an automatic construction of common-sense knowledge bases. It is a framework for adapting the weights of language models to learn to produce novel and diverse common-sense knowledge tuples (Bosselut et al., 2019). Abductive natural language inference can be used to interpret between the lines in natural language (Bhagavatula et al., 2019). Inferences allow us to connect pieces of knowledge to reach a new conclusion. Humans perform natural language inference based on a vast amount of external knowledge about language and the world. To comprehend human language, machines first need linguistic knowledge, i.e., knowledge about the language. This includes the understanding of word meanings, grammar, syntax, semantics, and discourse structure. Having linguistic knowledge gives a human or machine the basic capabilities of understanding language and virtually is a required property of any NLP system, even those not created for NLI tasks. Common knowledge refers to well-known facts about the world that are often explicitly stated (Cambria et al., 2011). This type of knowledge is often referred to human communication (Cambria et al., 2014). Some types of common knowledge may be domain-specific. While domain-specific knowledge is obviously useful for domain-specific applications, much of this knowledge may not be needed for general-purpose communication with humans. Common-sense knowledge, on the other hand, is typically unstated, as it is considered obvious to most humans and consists of universally accepted beliefs about the world. common-sense knowledge provides a deeper understanding of language. While it is rarely referred to language, humans rely on it in communication (Gao et al., 2016), as it is required to reach a common ground. It consists of everyday assumptions about the world and is generally learned through one's own experience with the world but can also be inferred by generalizing over common knowledge. While common knowledge can vary by region, culture, and other factors, we expect that common-sense knowledge should be roughly typical of all humans (Davis et al., 2017).

To tackle the challenging benchmark tasks, many computational models have been developed. These range from earlier symbolic and statistical approaches to recent approaches based on deep neural networks. Explicit textual content is used for different tasks, such as hate speech detection systems, and the primary challenge for statistical and neural classifiers is to infer the implicit messages in text. Recent studies have highlighted the need to use implicit messages to detect textual content (ElSherief et al., 2021). Knowledge graphs have been constructed to answer user questions by identifying the reasoning relations (Jin et al., 2023b). Similarly, an external knowledge base was used with the transformer to perform emotion recognition and bias prediction (Ghosal et al., 2020; Swati and Grobelnik, 2022). Semantic knowledge also proved to enhance the existing model to learn a general representation (Razniewski et al., 2021). There are many examples of recommendation systems that utilize semantic knowledge consisting of several attributes and multi-model knowledge (Zhou et al., 2020; Lei et al., 2021). Taking the semantic information through knowledge graphs is also one of the best ways to associate semantic information with the data for different tasks (Colon-Hernandez et al., 2021). Common-sense knowledge consists of many spatiotemporal features, including spatial, physical, temporal, and psychological aspects of everyday life. It has proven to be crucial for many NLP tasks, including dialogue understanding and generation, event prediction, and question answering (Fang et al., 2021). For the development of new approaches to address different tasks, one of the critical tasks is creating benchmark datasets to evaluate the approaches (Storks et al., 2019).

### 2.3. Sentiments as semantic knowledge

Sentiment classification of news deals with the identification of positive and negative news that can be used to predict trends related to different tasks (Yazdani et al., 2017). Sentiment of the news has already been used for news classification and other features, including entities and special phrases (Demirsoz and Ozcan, 2017; Hui et al., 2017). In the task of sentiment classification approaches, DistilBERT can transfer basic semantic understanding to further domains, and lead to greater accuracy than the baseline TFIDF (Dogra et al., 2021). For the task of fake news detection, the textual content of the news along with the headline has been used to extract the features (Cui et al., 2019). Taj et al. (2019) used dictionary-based and corpus-based methods for sentiment analysis of news related to business, entertainment, politics, sport, and technology. Li et al. (2017) have used sentiments along with a bag of features to predict the stock market prediction. Aspect-based sentiment analysis has been performed by infusing external background knowledge in the form of triples (Jin et al., 2023c). Bhutani et al. (2019) prove that sentiments of fake news increase the accuracy of fake news detection, and there exists a strong relationship between news and its sentiments, such as negative emotions tend to spread fast (Ajao et al., 2019).

## 3. Approach description

To perform the classification of news published across barriers (geographical, cultural, economic, etc.) and, in that attempt, to recommend and identify trends of news spreading belonging to different categories, some methodological considerations are necessary.

**Figure 1:**
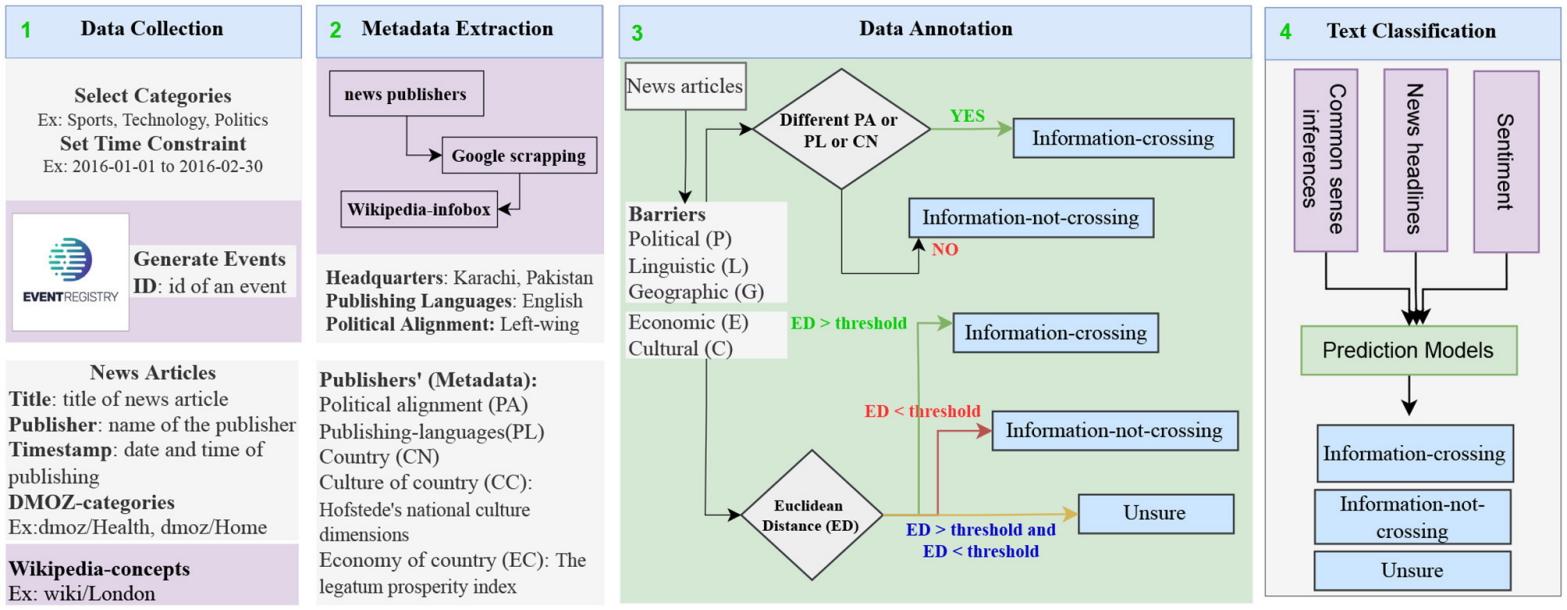
An approach to automatic barrier profiling based on the news meta-data. Data extraction from the Event Registry is the first step. Meta-data extraction through Google and Wikipedia scraping is the second step. The third step is to label the news articles after calculating the Euclidean distances. The classification with classical machine learning models, deep learning, and transformer-based methods is performed in the last step.

This research article presents a novel approach to barrier annotation utilizing news meta-data and an approach to news classification utilizing inference-based semantic knowledge, as shown in Figure 1. In the first step, we execute a query that extracts the news articles from the Event Registry belonging to different categories (business, computers, games, health, home, recreation, science, shopping, society, and sports) and publishes them within a certain time span – in our case, between 2016 and 2021 (see Section 4). Then, we parse and save these news articles along with the source information, such as publishers' names and publishing dates.

In the second step, we extract meta-data related to news publishers *via* searching the news publishers' on Google and extracting their Wikipedia links. Using these links, we obtain the necessary information from Wikipedia-Infobox (Sittar et al., 2022b). We use the Bright Data service^1^ to crawl and parse Wikipedia-Infobox.

In the third step, we perform the annotation of news articles. To label the news articles, we set the annotation guidelines (see Section 4). For cultural and economic barriers, we assign ternary labels to news articles, whereas for linguistic, geographical, and political barriers, we assign binary labels to news articles.

**Figure 2:**
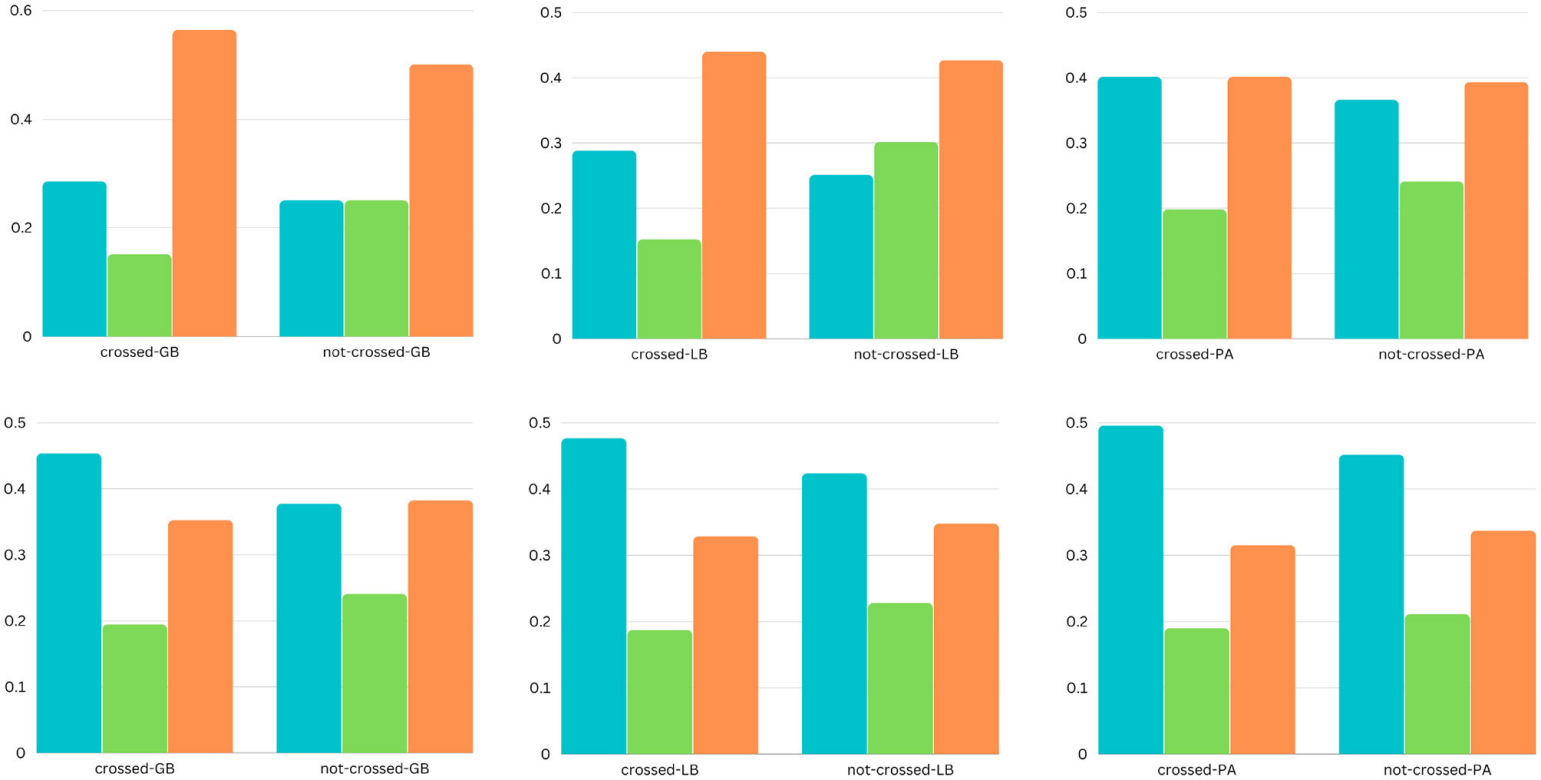
The bar charts present the comparison of sentiments (positive, neutral, and negative) in the headlines for two categories (shopping and society). Each column represents (from top to bottom) the two categories, and each row represents (from left to right) the two barriers (cultural and economic).

**Figure 3:**
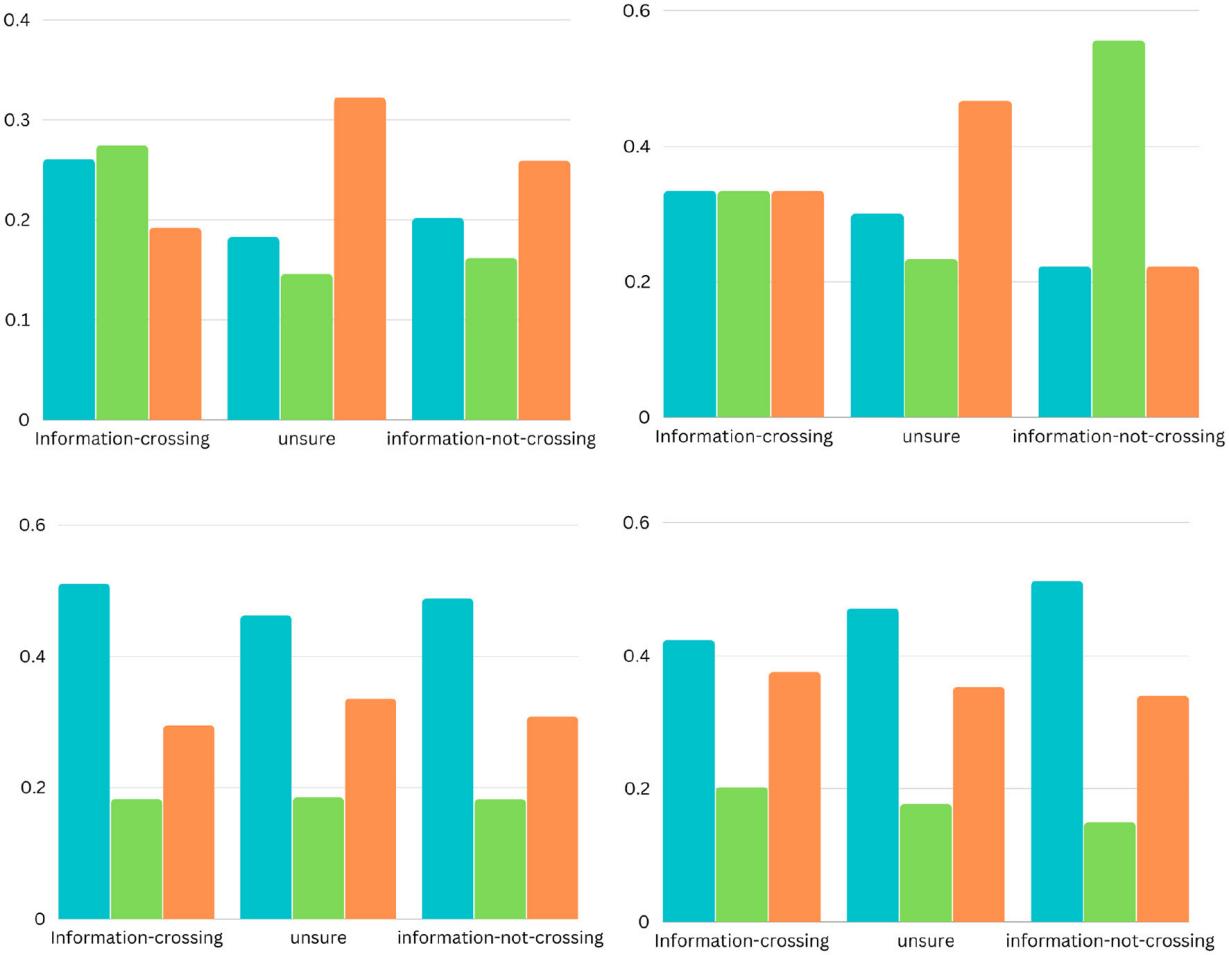
The bar charts present the comparison of sentiments (positive, neutral, and negative) in the headlines for two categories (shopping and society). Each column represents (from top to bottom) the two categories, and each row represents (from left to right) the three barriers (geographic, linguistic, and political).

**Figure 4:**
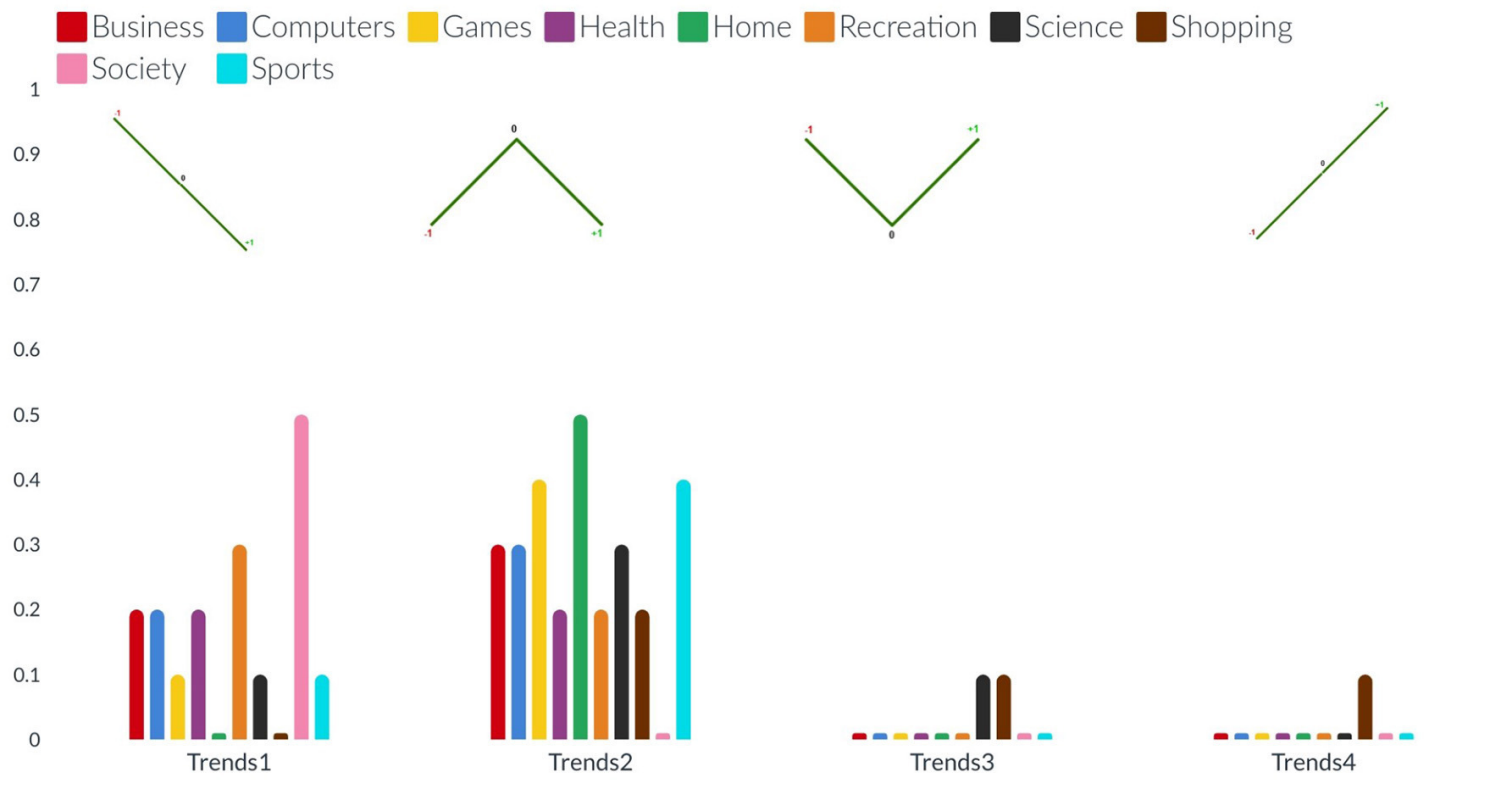
The bar charts present the distribution of different possible trends of sentiments across the ten categories (from left to right). The sentimental trends vary in four different types (see on the x-axis): trend1, and trend4 represent decrement and increment respectively in the percentage of news articles (see on the y-axis) with negative sentiment to neutral and then to positive: trend2, and trend3 represent decrement and increment respectively in the percentage of news articles with neutral sentiments than positive and negative sentiments.

**Figure 5:**
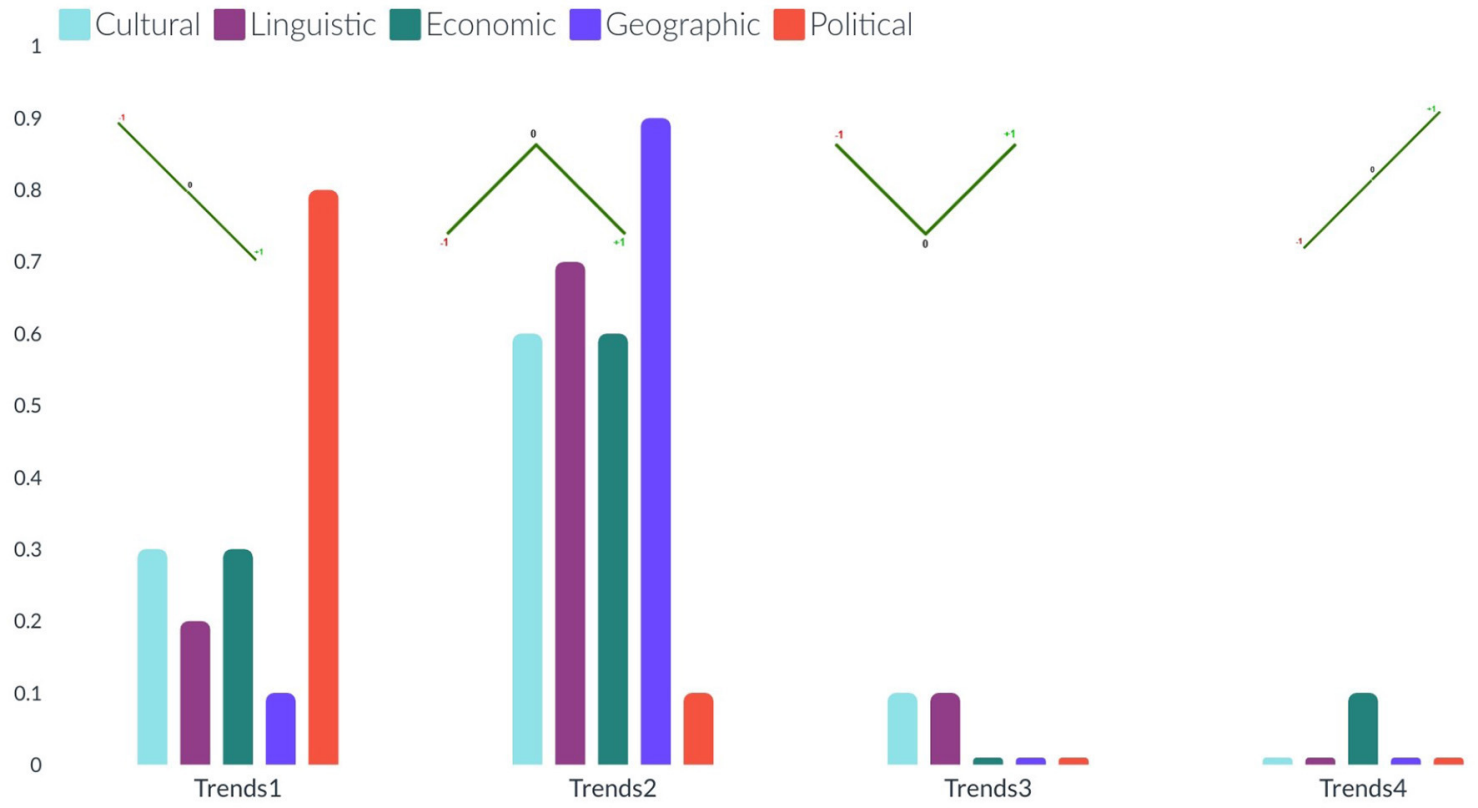
The bar charts present the distribution of different possible trends of sentiments across the five barriers (from left to right). The sentimental trends vary in four different types (see on the x-axis): trend1, and trend4 represent decrement and increment respectively in the percentage of news articles (see on the y-axis) with negative sentiment to neutral and then to positive: trend2, and trend3 represent decrement and increment respectively in the percentage of news articles with neutral sentiments than positive and negative sentiments.

**Figure 6:**
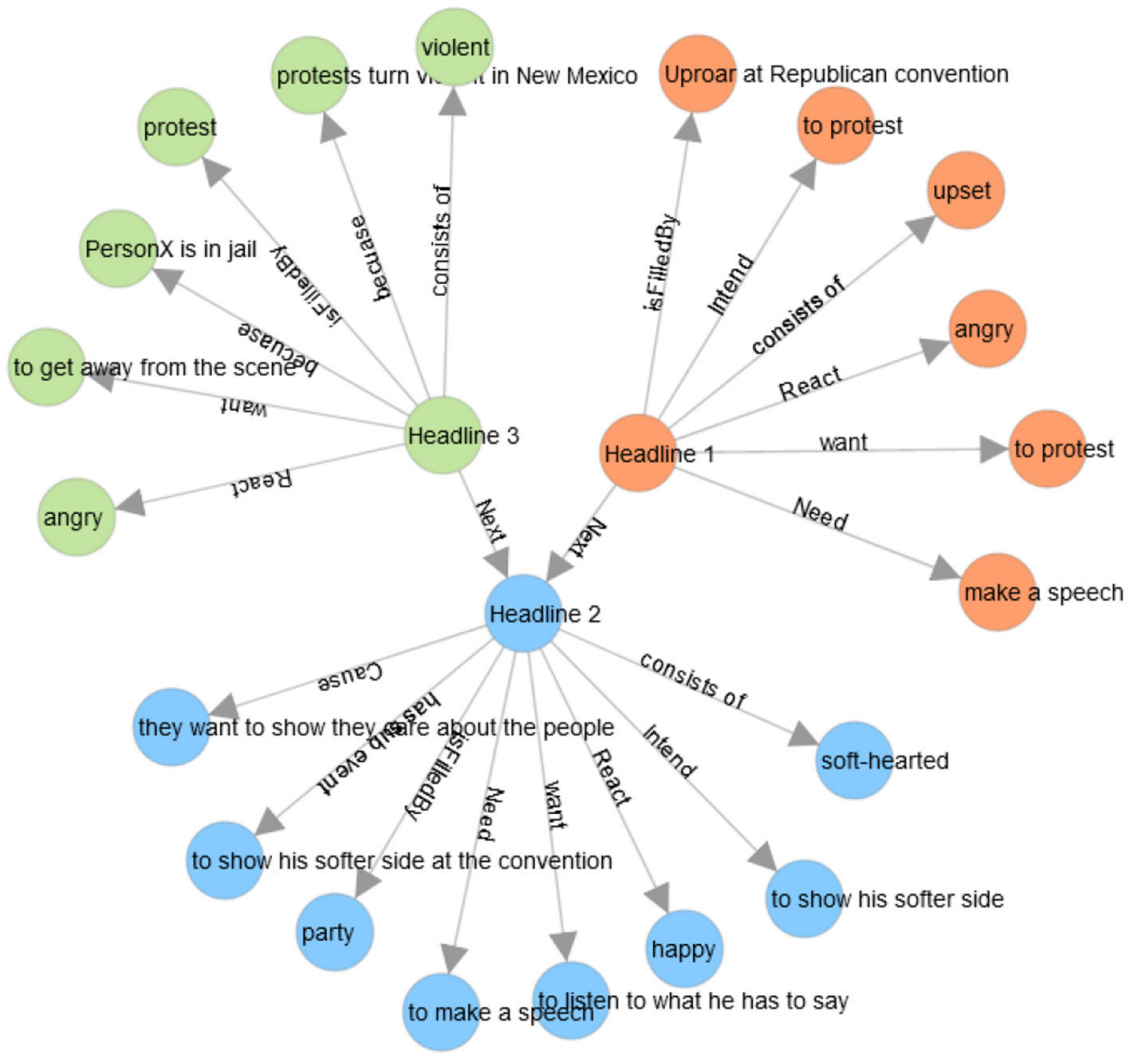
The network diagram presents an example of headlines with common-sense knowledge. Headline 1 is “Uproar at Republican convention as anti-Trump delegates revolt,” Headline 2 is “Trump aims to show his softer side at Cleveland convention,” and Headline 3 is “Protests turn violent outside Trump rally in New Mexico” [these headlines belong to the cultural barrier (see Table 2)].

**Figure 7:**
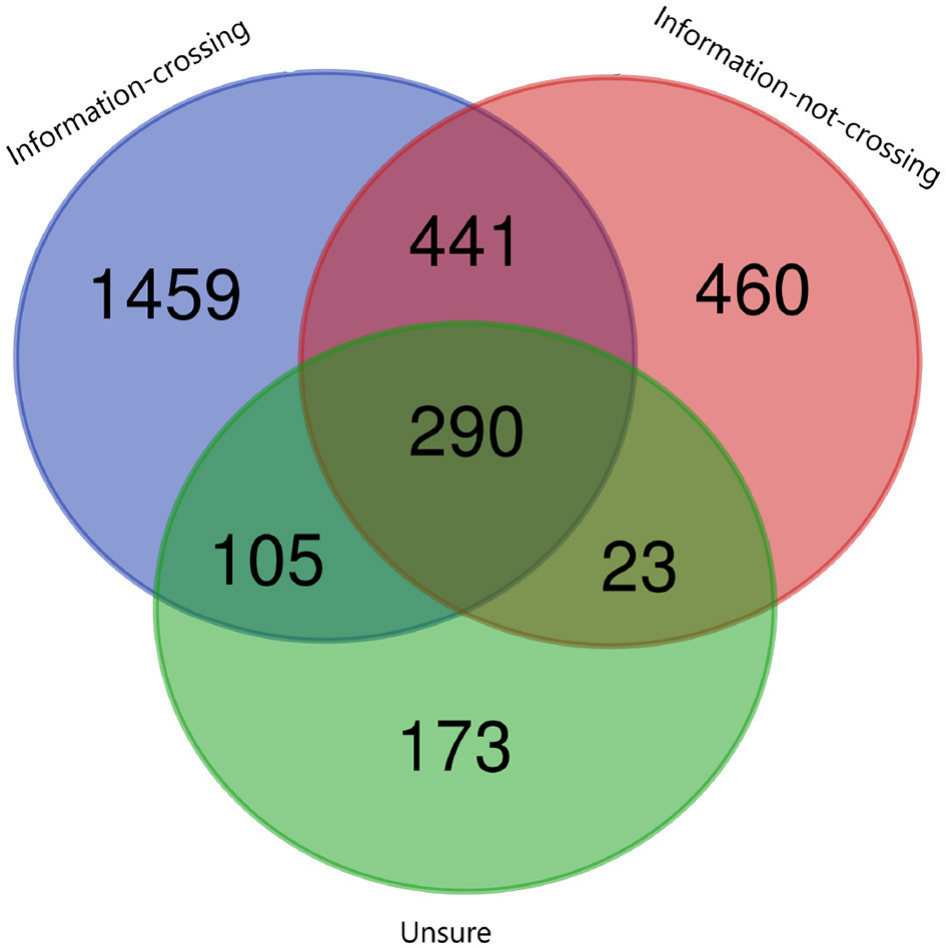
The Venn diagram shows the intersection between inferences across ternary classes of the cultural barrier.

**Figure 8:**
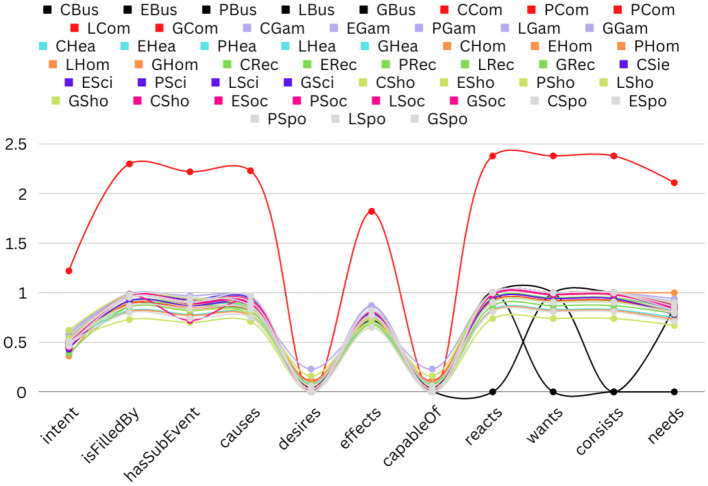
The line graph shows the frequency of all the inferences to all categories of all the barriers. The list of inferences has been shown on the x-axis, whereas the average number of inferences per news headline has been shown on the y-axis.

In the fourth step, we conduct a detailed analysis of the sentiments of the news headlines for each category (see Figures 2, 3) and provide a list of comprehensive trends of sentiments across different categories and barriers (see Figures 4, 5). Next, we extract semantic knowledge through the inference-based model COMET (Bosselut et al., 2019) (see Figure 6). We analyze the properties of the relations to the news headlines of different topics (see Figures 7, 8). Afterward, we conduct experiments comparing machine learning state-of-the-art (LR, NB, SVC, kNN, and DT), deep learning (LSTM), and transformer-based methods (BERT) using a combination of news headlines with inference-based semantic knowledge and its sentiments. The results are presented in Sections 6.1 and 6.2 showing the performance of different features and methods. The source code for this approach is available in the GitHub repository.^2^

More specifically, we focus on the following research questions:

1. RQ1: Do the sentiments of the news headlines on different topics vary across the different barriers?
2. RQ2: What are the properties (statistics and ratio) of the common-sense knowledge relations in news headlines to different topics?
3. RQ3: Which classification methods (classical or deep learning methods or transformer-based methods) yield the best performance to barrier classification task?

## 4. Benchmark dataset construction

We collected the news articles reporting on different events published between 2016 and 2021 in the English language using Event Registry (Leban et al., 2014) APIs.^3^ The dataset consists of approximately 1.7 million news articles. Each news article belongs to a different category (see Table 1). Each news article consists of a few attributes, such as title, body text, name of the news publisher, date and time of publishing, event-ID, DMOZ-categories, and Wikipedia concepts.

**Table 1:** Statistics of the news articles based on common-sense knowledge extraction and data annotation for the 10 categories (business, computers, games, health, home, recreation, science, shopping, society, and sports) of the five barriers.

**Categories**	**Cultural**	**Economic**	**Geographic**	**Linguistic**	**Political**
**Business**	3,455	1,015	3,550	7,974	7037
**Computers**	913	310	1,181	2,194	1895
**Games**	186	68	549	504	316
**Health**	1,159	90	1,533	3,295	3368
**Home**	1,065	86	1,321	2,796	3258
**Recreation**	1,695	161	1,783	3697	3236
**Science**	4,377	378	7,877	14,665	14925
**Shopping**	513	42	796	1,685	1287
**Society**	14,238	1,003	13,472	28,431	28447
**Sports**	1,533	39	1,021	2,054	1289

A few attributes are self-explanatory, such as title, body text, name of the news publisher, and date and time of publication. An event-id represents a unique number that is associated with all the news articles that belong to the same event. The DMOZ-categories represent the topics of the content or news article. It is a project that has a hierarchical collection of web page links organized by subject matter.^4^ Approximately 50,000 categories are used by the Event Registry (top 3 layers of the DMoz taxonomy).^5^ The statistics of all the categories for all five barriers are presented in Table 1. The Wikipedia concepts are used as a semantic annotation for news articles and can represent entities (locations, people, or organizations) or non-entities (things such as personal computers and toys). In the Event Registry, Wikipedia's URLs are used as concept URIs.

**Figure 9:**
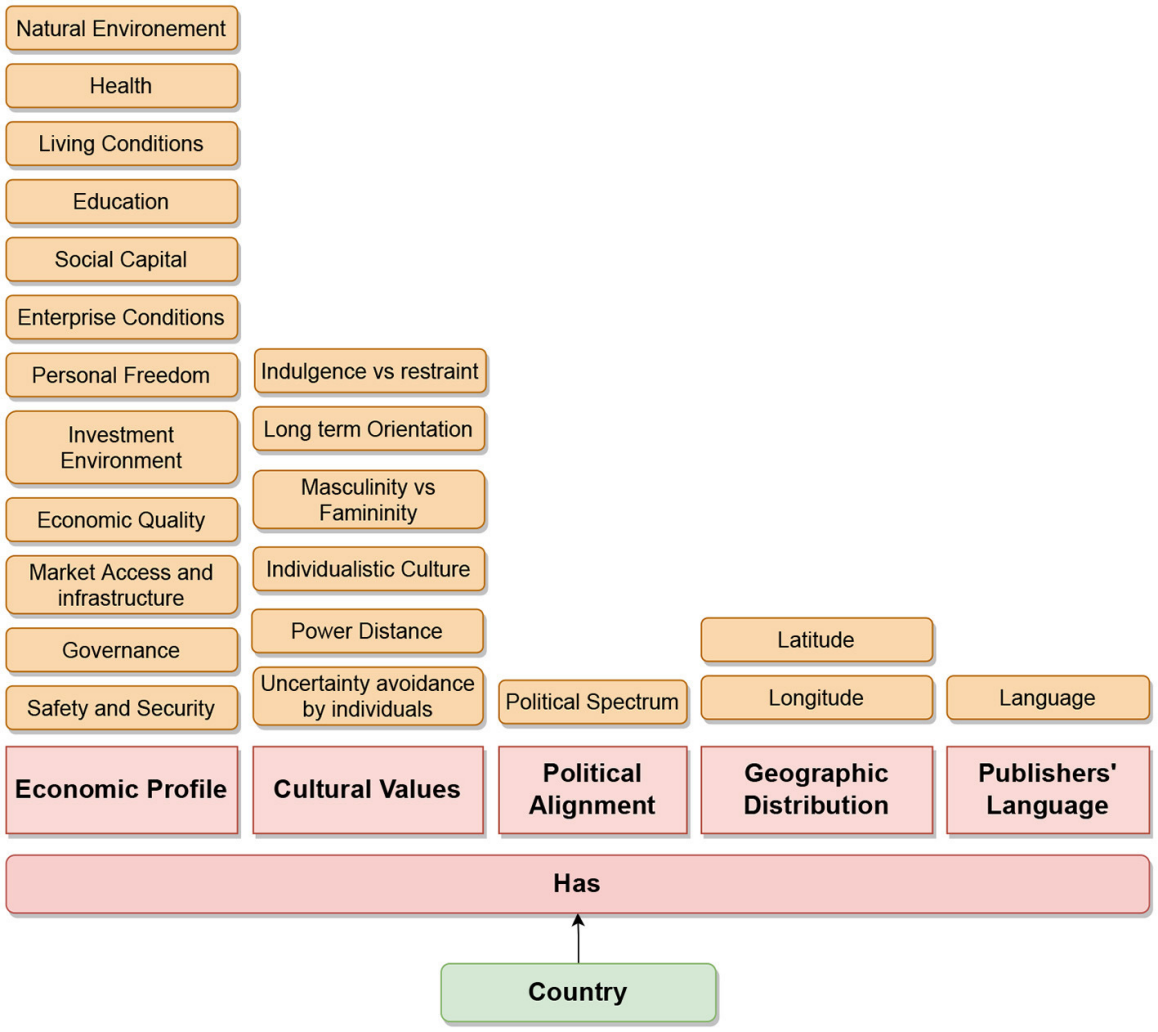
Metadata for the five barriers (cultural, economic, geographical, linguistic, and political).

**Figure 10:**
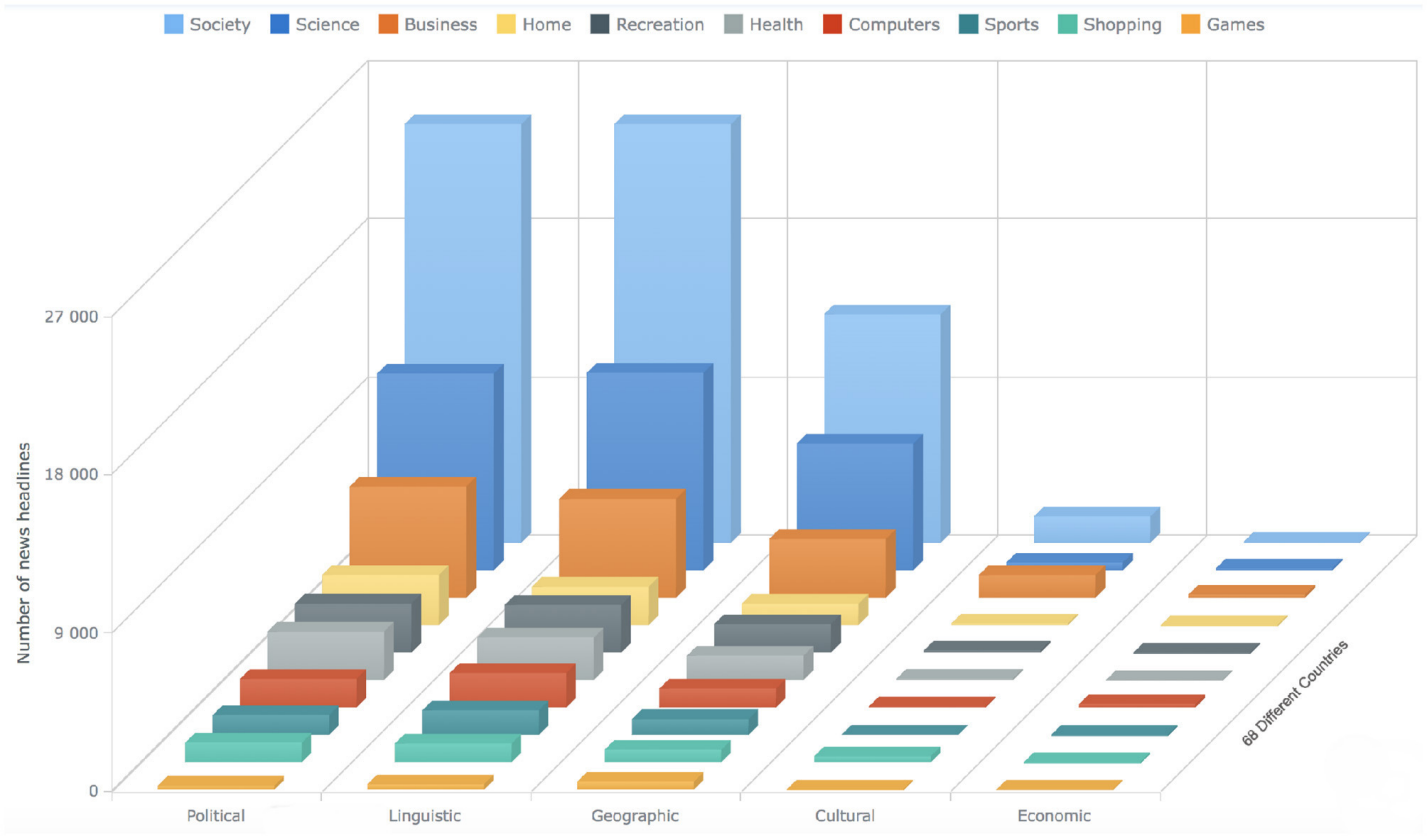
The bar chart shows the statistics about the news articles that has the label “Information-crossing” for all the 10 different categories. The x-axis shows the different barriers, the y-axis shows the count of the news articles, whereas the bars on the z-axis represent 10 different categories (see Section 4).

**Figure 11:**
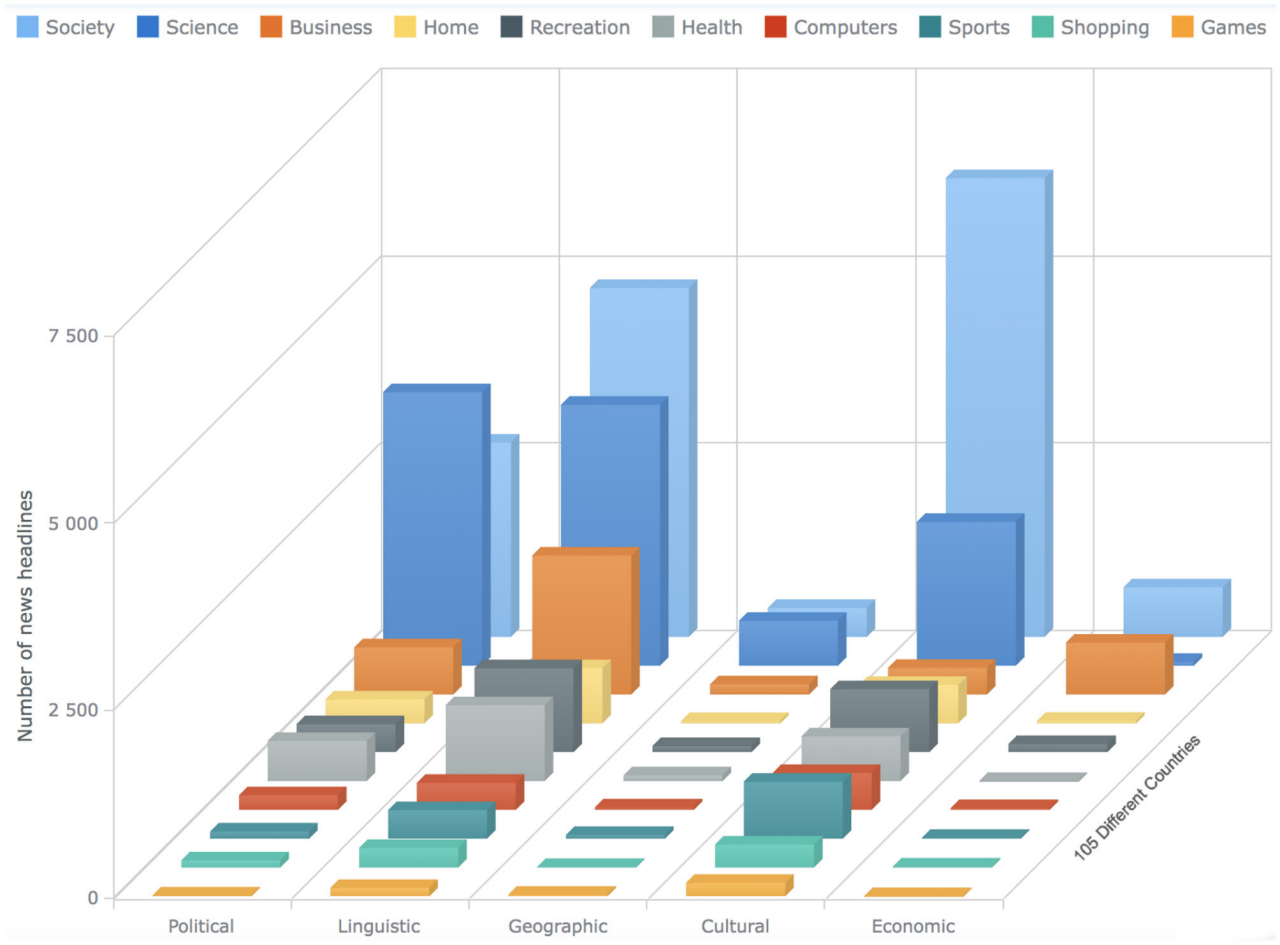
The bar chart shows the statistics about the news articles that has the label “Information-not-crossing” for all the 10 different categories. The x-axis shows the different barriers, the y-axis shows the count of the news articles, whereas the bars on the z-axis represent 10 different categories (see Section 4).

**Figure 12:**
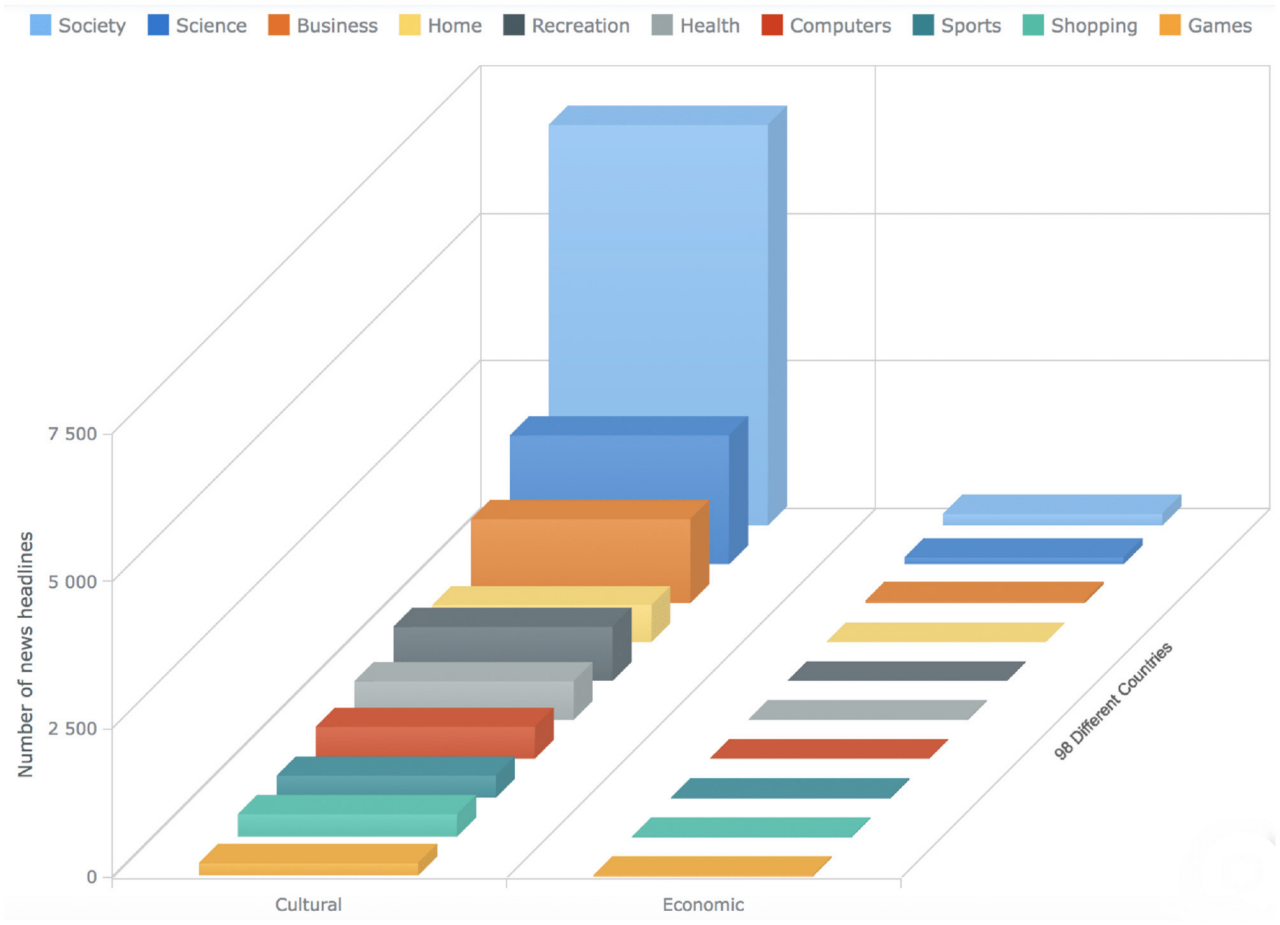
The bar chart shows the statistics about the news articles that has the label “Unsure” for all the ten different categories. The x-axis shows the different barriers, the y-axis shows the count of the news articles, whereas the bars on the z-axis represent ten different categories (see Section 4).

To fetch the metadata for each barrier, the essential thing is the news publisher's headquarter name (see Figure 9). For each news publisher, we get this information from Wikipedia-Infobox. We used the Bright Data service (see text footnote 1) to crawl and parse Wikipedia-Infobox for almost more than 10,000 news websites. We retrieved the country name of the news publisher's headquarters. For the economical barrier, we fetched the economical profile for each country using “The Legatum Prosperity Index”^6^ as done by Sittar et al. (2022b). It has 12 dimensions that represent different economic aspects. For the cultural barrier, we calculated differences among different regions using six Hofstede's national culture dimensions (HNCD). For the economic and cultural barriers, we calculated the Euclidean distance among all the countries (for the economic barrier using the economical profile and for the cultural barrier using the HNCD). Two countries were labeled as “information-not-crossing” if the distance score was ≤ 0.1, “unsure” if the distance score was >0.1 and ≤ 0.4, and “information-crossing” if the distance score was >0.4. For the geographical barrier, we stored general latitude and longitude. For the political barrier, we utilized the political ideology or alignment of the newspaper or magazine that we determined based on Wikipedia-Infobox at their Wikipedia page (Sittar et al., 2022a). The barriers, including cultural, economic, and geographic, do not have a standard representation. They have been estimated by utilizing a relevant set of features. In case of the political and linguistic barriers, we utilized the available political alignments and publishing languages from the Wikipedia-Infobox of a specific publisher. The statistics about the labeled dataset are presented in Figures 10–12 and Table 1. Data can be found in the GitHub repository (see text footnote 2).

We set the following annotation questions based on the definitions mentioned above in order to classify the barriers to news spreading.

Q1: *Do all the news articles reporting on an event publish from a particular or the same geographical location?*
Q2: *Do all the news articles reporting on an event publish from the locations having equal economic prosperity?*
Q3: *Do all the news articles reporting on an event publish from a particular or thesame locations having equal cultures?*
Q4: *Do all the news articles reporting on an event publish from sources with a particular or similar political class?*
Q5: *Do all the news articles reporting on an event publish by the newspapers where the publishing language was same?*

Question 1 (Q1) intends to identify whether the news was published across different geographical places or not. The question is answered “yes” for all the news articles reported on an event if they are published in one country otherwise “no.” Question 2 (Q2) intends to identify whether the news was published across different economies or not. The economic similarity has been calculated using Euclidean distance. The question is answered with “information-crossing” for all the news articles reported on an event if they are published from countries with similar economic situations. The question is answered with “unsure” for all the news articles reported on an event if at least one of the news articles is published from a country that is labeled with “unsure” otherwise “information-not-crossing.” Question 3 (Q3) intends to identify whether the news was published across different cultures or not. The question is answered with “information-crossing” for all the news articles reported on an event if they are published from countries with a similar culture. The question is answered with “unsure” for all the news articles reported on an event if at least one of the news articles is published from a country that is labeled with “unsure” otherwise “information-not-crossing.” The cultural similarity has been calculated using the Euclidean distance (see Section 4). Question 4 (Q4) intends to identify whether the news was published in newspapers with the same political alignments or not. The question is answered “yes” for all the news articles reporting on an event if they are published in the newspapers following similar political alignments, otherwise “no.” Question 5 (Q5) intends to identify whether the news was published in the newspapers where the publishing language was the same or not. The question is answered “yes” for all the news articles reporting on an event if they are published from different newspapers, where the publishing language is the same otherwise “no.”

### 4.1. Annotated dataset

Initially, we collected approximately 1.7 million news articles. After filtering the news based on the unavailability of the metadata information, the news articles were limited to a few thousand articles. Similarly, based on not having any common sense inferences, the news articles were reduced to a few thousand articles. The number of news articles was reduced from 75 to 96%. The statistics of the news belonging to the 10 categories across the five barriers are presented in Table 1. The dataset is available in the GitHub repository (see text footnote 2). Labels for annotations of the five types of barriers are derived as follows:

- Economic barrier classes: *information-not-crossing, unsure*, and *information-crossing*.
- Cultural barrier classes: *information-not-crossing, unsure*, and *information-crossing*.
- Geographical barrier classes: *Not-crossed-GB* and *Crossed-GB*.
- Political barrier classes: *Not-crossed-PB* and *Crossed-PB*.
- Linguistic barrier classes: *Not-crossed-LB* and *Crossed-LB*.

## 5. Materials and methods

In this section, we present an analysis of sentiments across different barriers, followed by the properties of common-sense inference knowledge, classification baselines, and evaluation metrics.

### 5.1. Analysis of sentiments

We use the Vader rule-based model to obtain the emotional and sentiment polarity of the news headlines to analyze the variation of sentiments across the different categories of the different barriers. Vader provides a polarity range for the news headlines in the interval from –1 to +1. The –1 value represents a negative polarity, and +1 indicates a positive polarity (Mart́ın et al., 2021). The bar charts illustrate the differences in sentiments across binary and ternary classes in two categories of the three barriers (see Figures 2, 3). For each instance, we have one of the three sentiments, such as positive, neutral, or negative.

For the binary class classification of the political, linguistic, and geographical barrier, the headlines that have been labeled as crossing the barrier have the following sentimental differences: The categories business, home, health, recreation, science, shopping, and society have more instances of negative sentiments than positive and neutral, with considerable differences of (8, 1, 10, 5, 8, 5, and 5%), (6, 5, 2, 5, 8, 5, and 5%), and (7, 12, 4, 12, 9, 3, and 5%), respectively. The game category of news headlines with annotations of crossing the political barrier has 12, 6, and 1% more instances of positive sentiments for the political, linguistic, and geographical barriers; the news headlines that have been labeled as not crossing the political barrier have the following differences: the categories computers, health, recreation, science, society, and sports have more instances of positive sentiments than neutral and negative sentiments, with considerable differences of (5, 8, 5, 5, 2, and 5%), (7, 2, 5, 5, 2, and 3%), and (4, 2, 1, 8, 3, and 5%), respectively; the game category has 10, 1, and 3% more instances, respectively, with negative instances than other classes; With regard to the ternary classification of the cultural barrier, the news headlines that have been labeled as crossing the barrier have the following sentimental differences: the categories games, health, shopping, and society have more instances of negative sentiments than other classes, with considerable differences of 12, 5, 6, and 2% respectively. The news headlines that have been labeled as not crossing the cultural barrier have the following differences: the categories business, computers, health, recreation, science, shopping, society, and sports have more instances of positive sentiments than other classes with considerable differences of 5, 6, 5, 5, 10, 3, and 8%, respectively. The news headlines that have been labeled as unsure have the following differences: the categories computers and shopping have more instances of positive sentiments than other classes, with considerable differences of 6 and 5%, respectively, whereas the category game has 20% more instances of negative sentiments; with regard to the ternary classification of the economic barrier, the headlines that have been labeled as crossing the barrier have the following sentimental differences: the categories business, home, recreation, science, shopping, and sports have more instances of negative sentiments than other classes, with the considerable differences of 5, 7, 8, 5, 2, and 14%, respectively. The news headlines that have been labeled as not crossing the economic barrier have the following differences: games, home, recreation, science, and shopping have more instances of positive sentiments than other classes, with considerable differences of 18, 16, 7, 5, and 8% respectively. The headlines that have been labeled as unsure have the following differences: The categories business and games have 5 and 22% respectively, more instances of negative sentiments, computers, and sports have 8 and 10% respectively more instances of positive sentiments; and recreation and shopping have 12 and 13% more instances of neutral sentiment, respectively.

Overall, with regard to the binary class classification for the political, linguistic, and geographical barriers, we see that the news headlines that are labeled as crossing the barrier, have more instances of negative sentiments, whereas the news headlines that are labeled as not crossing the barrier, have more instances of positive sentiments. With regard to the ternary class classification for the economic and cultural barrier, we see that the news headlines that are labeled as crossing the barrier have more instances of negative sentiments, whereas the news headlines that are labeled as not crossing the barrier have more instances of positive sentiments. However, in the case of news headlines that are labeled as unsure, there are more instances of negative sentiments for the economic barrier and positive sentiments for the cultural barrier.

The bar charts present the distribution of different possible trends of sentiments across the ten categories and five barriers (see Figures 4, 5). The purpose of this figure is to show the investigation that we did while finding the variations of sentiments across different categories and barriers. It tells the readers what type of news has more positive, neutral, or negative polarity. It especially helps us across the barriers to know what type of barriers are crossing positive or negative news. We analyzed the sentimental trends and found that, among other possible trends, these four trends cover more than 95% of the data. The first trend shows that the number of positive instances is higher than the number of neutral instances, and then both are higher than the number of negative instances. The fourth trend is the reverse of it. The second trend shows that the number of neutral instances is higher than that of positive and negative instances, and negative and positive instances are approximately equal to each other. The first bar chart shows that more than 30% of news of society and recreation categories consist of news headlines with positive sentiment, whereas more than 30% of news of games, home, and sports categories consist of news headlines with neutral sentiments. The second line graph shows that 80% of news headlines belonging to the political barrier have negative sentiment, whereas ninety 90% of news headlines belonging to the geographical barrier have neutral sentiments.

The results suggest the following conclusions for the **Q1**: (1) The political barrier has been crossed by the news with positive sentiments and reversed for the other four barriers (linguistic, geographic, cultural, and economic). The news with negative sentiments has not been crossing the political barrier but has been crossing the linguistic, geographic, cultural, and economic barriers; (2) the variations in the sentiments across binary and ternary class classifications of the different categories of news and the barriers suggest that we should take sentiment score as a feature in barrier classification. Alonso et al. (2021) have considered sentiments of news for fake news detection based on the fact that sentiment is a complementary element to fake news.

### 5.2. Common-sense knowledge

We use the common sense knowledge resource COMET atomic through an inference toolkit called *kogito* to generate common-sense inferences in a given situation by assessing their intentions and behaviors. This toolkit provides the interface to interact with natural language generation models that can be used to infer common-sense from a textual input (Hwang et al., 2021; Ismayilzada and Bosselut, 2022). These models consist of triplets of head entity, relation, and tail entity. We present an example illustrating the results of the common-sense of different relations about three news headlines (taken from the Table 2). The first headline (“Uproar at Republican convention as anti-Trump delegates revolt”) has six relations such as *react, need, intend, want, isFilledBy, and react*. To convert common-sense knowledge into a meaningful text, we consider each tuple consisting of the relation and tail as a sentence and then concatenate them. To make the tuples as sentences, we change the relation to the past form, such as reacted angry, needed to make a speech, intended to protest, wanted to protest, isFilledBy uproar at the Republican Convention, and reacted upset.

**Table 2:** Examples of annotation for all five types of barriers.

**Barrier (Category)**	**Time**	**Title**	**Location/Publisher/Language**	**Meta-data**	**Class**
**Cultural** **(Games)**	2016-07-18T19:48:00Z 2016-07-18T22:04:00Z	Trump aims to show his softer side at Cleveland convention Uproar at Republican convention as anti-Trump delegates revolt	Ireland (irishtimes.com) Thailand (bangkokpost.com)	Same Culture	Information -not-crossing
	2016-04-16T22:23:00Z 2016-04-18T16:02:00Z	Another small victory for Cruz Romney: 3-man race throws Trump the nomination	New Zealand (odt.co.nz) United States (wcyb.com)	Different Culture	Unsure
	2016-05-25T05:19:00Z 2016-05-25T11:40:00Z	Protests turn violent outside Trump rally in New Mexico Protests turn violent outside Trump rally in New Mexico	japan (japantoday.com) United States (newschannel5.com)	Different Culture	Information -crossing
**Economic** **(Recreation)**	2016-07-13T21:36:00Z 2016-07-16T19:59:00Z	File to seek Gulen's US extradition ready Erdogan calls on Barack Obama to extradite Fethullah	Azerbaijan (en.trend.az) Armenia (news.am)	Similar economic Situations (ES)	Information -not-crossing
	2018-03-17T20:46:00Z 2018-03-19T17:14:00Z 2018-03-22T01:50:00Z	Trump consultants harvested data from 50 million Facebook users: reports Officials question Facebook's protection of personal data Ex-Facebook manager says company was sluggish in stopping data harvesting	Pakistan (geo.tv) United States (union-bulletin.com) Kenya (businessdailyafrica.com)	Different ES	unsure
	2018-04-03T17:19:00Z 2018-04-04T18:13:00Z	Trump seeks Syria pullout as advisers warn on Islamic State White House appears to delay Trump's order for Syrian withdrawal	Egypt (english.ahram.org.eg) Iraq (kurdistan24.net)	Different ES	Information -crossing
**Political** **(Society)**	2021-04-07T21:55:00Z 2021-04-08T06:52:00Z 2021-04-09T03:22:00Z	Thugs petrol bomb bus as violent riots continue in Belfast Thugs petrol bomb bus as violent riots continue in Belfast Police use water cannon during continued unrest in belfast	conservatism (thesun.ie) conservatism (thesun.ie) centre-right (theaustralian.com.au)	Similar political alignment (PA)	information -not-crossing
	2016-01-06T14:51:00Z 2016-01-08T01:49:00Z 2016-01-09T01:08:00Z	Iraq offers to mediate in Saudi-Iran crisis stemming from cleric's execution Iran not seeking tension with Saudi Arabia: Zarif Hammond fails to condemn Saudia political executions	Neutral (brandonsun.com) Conservatism (tehrantimes.com) Left-wing (morningstaronline.co.uk)	Different PA	information -crossing
**Linguistic** **(Society)**	2016-01-15T14:50:00Z 2016-01-15T17:51:00Z 2016-01-16T02:20:00Z	Why Amal Clooney doesn't think she's a celebrity Amal Clooney talks about her new celebrity status for the first time Amal Clooney sits down for First U.S. TV interview Watch!	English (pagesix.com) English (vanityfair.com) English (usmagazine.com)	Similar publishing Language (PL)	information -not-crossing
	2016-01-15T17:38:00Z 2016-01-15T14:00:00Z 2016-01-15T16:27:00Z	Friendly no more: Trump, Cruz erupt in bitter fight at Republican debate The fight everyone wanted to see finally happened The 6th republican debate in 100 words (and 4 videos)	Spanish (ecodiario.eleconomista.es) English (charismanews.com) English (scpr.org)	Different PL	information -crossing
**Geographic** **(Society)**	2021-04-28T11:48:00Z 2021-04-28T22:52:00Z	Lawmaker says schools must teach the “Good of slavery” (Video) Backlash on Louisiana lawmaker grows following his comments about slavery	United States (patheos.com) United States (theadvertiser.com)	Publishers' headquarters in same country	information -not-crossing
	2016-06-10T11:21:00Z 2016-06-10T14:29:00Z 2016-06-10T15:18:00Z	Queen's dedication praised at 90th Queen's dedication praised at 90th Queen's dedication praised at 90th	England (haverhillecho.co.uk) United Kingdom (newsletter.co.uk) Australia (adelaidenow.com.au)	Publishers' headquarters in different country	information -crossing

The annotation is performed using the meta-data.

The purpose of using semantic knowledge in the form of common-sense knowledge was to improve text classification. We analyzed the associated inferences to all the barriers. We present an example to illustrate the comparison. We choose the cultural barrier, and to perform a comparison between the categories, we select the category of society. The results of the intersection between the inferences belonging to three different classes (information crossing, information not crossing, and unsure) have been shown in Figure 7. There are 290 inferences that are common among all three classes, and there are 441, 105, and 23 inferences that are common between classes one and two, class two and three, and class one and three, respectively. The most important fact is that there are 1,459, 173 and 460 unique inferences for classes one, two, and three, respectively, that can be useful for the classification in this ternary class classification.

To answer the **Q2** the line graphs in Figure 8 present the statistics of inferences across the ten different categories of the five barriers. Since the main purpose of this figure is to analyze the statistics of the inferences across the categories, we keep the same color for a category across the five barriers. The x axis shows the names of all the inferences, and the y axis shows the average number of inferences per news headline in each category. The categories that have significant differences are computer and business. The average inferences are significantly higher in the computer category than in all other categories. Each news headline contains 1.5% inference type “intent” and consists of “isFilledBy,” “hasSubEvent,” “Causes,” “reacts,” “wants,” “consists, and “needs” inference types with an average of approximately 2.5. The existence of the inference type is almost 0% per news headline for “desires” and “capableOf”. For the category business, the existence of a few types of inferences is equal to zero, such as “reacts,” “wants,” “consists,” and “needs.” Otherwise, the average of the existence of all the inferences per news headline is approximately equal for all the categories of all the barriers.

This analysis helps us to understand the distribution of different inferences across different categories, as well as the associated semantic knowledge per news headline. Since different features, such as sentiments and semantic knowledge, possess different discriminative capabilities in classification (Zhai et al., 2011; Nassirtoussi et al., 2015), we use them as classification of barriers to the spreading of news.

### 5.3. Evaluation methodology and baselines

**Figure 13:**
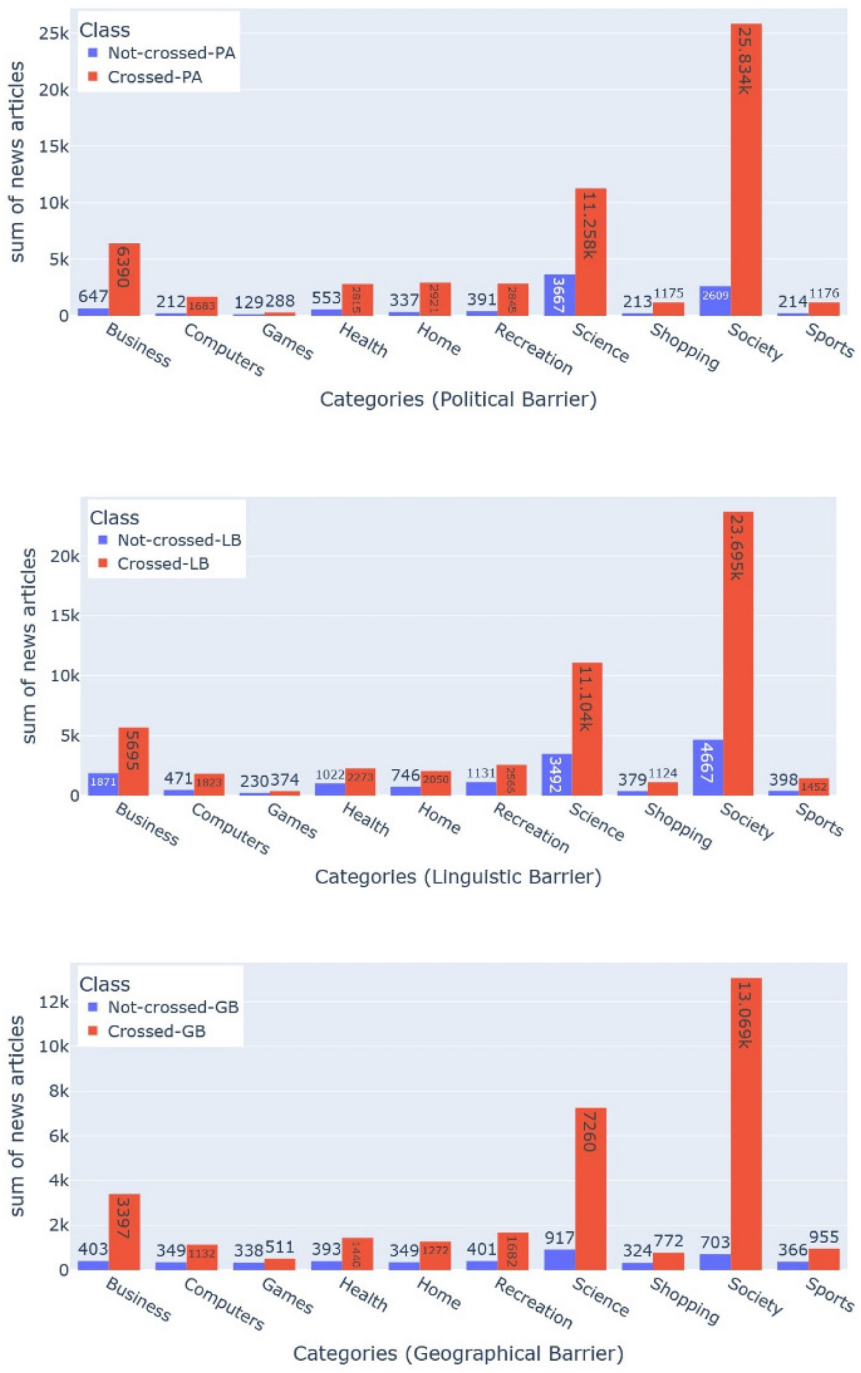
This bar charts show the class distribution for the political, linguistic, and geographical barriers (from left to right). The bar with blue color shows the distribution for the class “Information-crossing” a barrier, whereas the bar with orange color shows the distribution for the class “Information-not-crossing” a barrier. Each of the three-bar charts presents the class distribution for all ten categories.

**Figure 14:**
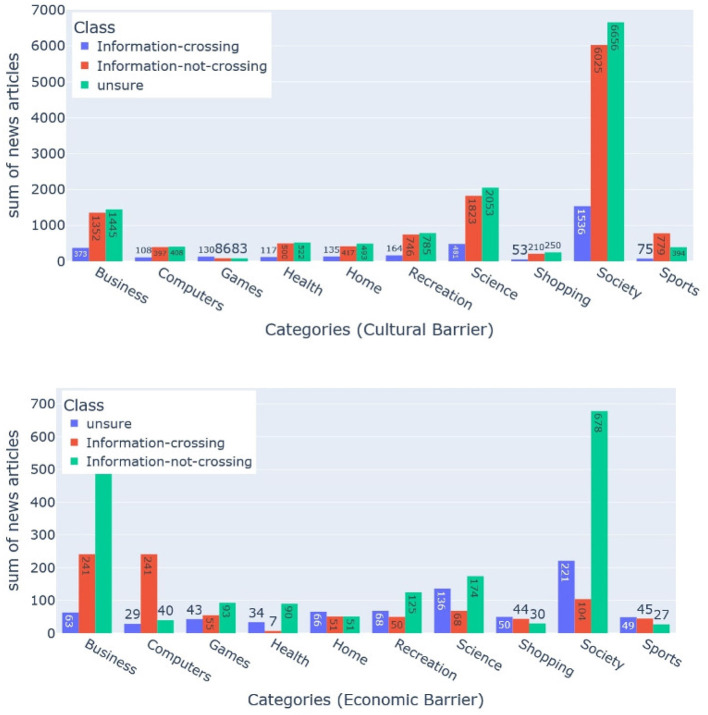
This bar chart shows the class distribution for economic, and cultural barriers (from left to right). The bar with orange color shows the distribution for the class “Information-not-crossing” whereas the bar with green color shows the distribution for the class “Unsure” a barrier. The bar with the blue color shows the distribution for the class “Information-crossing.” Each of the two bar charts presents the class distribution for all ten categories.

We used the Scikit-learn implementation of classical and deep learning models, considering the following parameters, which are usually the default: hidden layers = 3, hidden units = 64, no. of epochs = 10, batch size = 64, and dropout = 0.001. We provide the pseudocode of the classification models along with the features in Algorithm 1 and present a detailed description of each component. For the training process of the political, geographical, and linguistic barrier, we used Adam as the optimizer, binary cross-entropy as the loss function, and sigmoid as the activation function. For economic and cultural barriers, we used Adam as the optimizer, categorical cross-entropy as the loss function, and SoftMax as the activation function. The data about each barrier is split into train-sets and test-sets with a ratio of 80–20%. To maintain the class proportion in the train and test sets, we use stratified sampling. It means that the training and testing sets must have an equal proportion of all the classes. The Figures 13, 14 show the class distribution for each category of a barrier. For instance, in the case of the business category of the culture barrier, there are a total of 100 news headlines where 40 instances have the label “information crossing,” 40 instances have label “information not crossing,” and 20 instances have the label “Unsure.” And, we suppose, we split our train and test sets in the ratio of 80:20. Then, the train set and test set must have 20, 20, and 10 instances of each label (“information crossing,” “information crossing,” and “Unsure”) respectively.

**Algorithm 1:** Barrier classification algorithms—PM-LSTM and PM-BERT.

**Input** a set of news headline H **Output** predicted label: information-crossing or information-not-crossing or unsure
Find the sentiment S of a news headline (see Section 5.1) 1: for A headline in H **do**
2: *S*_*A*_ = *VADER*(*A*)
3: end **for**
Extract inferences-based semantic knowledge K in form of tuples (see Section 5.2) 1: for A headline in H **do**
2: *K*_*A*_ = *COMET*(*A*)
3: for each relation r in *K*_*A*_ **do**
4: if *r* is not in (*react, need, intend, want, isFilledBy, and react*) **then**
5: ignore r
6: end **if**
7: end **for**
8: end **for**
Convert the K tuples into sentences(see Section 5.2).
Calculate the feature vectors of H, K, and S and merge them together (see Section 5.3)
Hyper-parameter settings and training of the models 1: if *classes* = =*binary* **then**
2: *activation* = *sigmoid*
3: *optimizar* = *adam*
4: *loss*= binary cross entropy
5: else **if** *classes* = =*ternary* **then**
6: *activation* = *softmax*
7: *optimizar* = *adam*
8: *loss*= categorical cross entropy
9: end **if**
Evaluation on test data using F1-score (see Section 5.4)

For comparison with the proposed common sense inferences and semantic knowledge, we evaluated the barrier classification task using the news headline text only. After performing the preprocessing steps such as lower case conversion and stop word removal, we adopted the term frequency (TF) and inverted document frequency (IDF) methods to represent the bag of words in each news article (Yazdani et al., 2017). For the barrier classification task, the experiments were conducted by utilizing three different types of machine learning algorithms: (1) classical machine learning algorithms, including Logistic Regression (LR), Naive Bayes (NB), Support Vector Classifier (SVC), k-nearest Neighbor (kNN), and Decision Tree (DT): The performance of LR for text classification problems is the same as that of the SVM algorithm (Shah et al., 2020). SVMs use kernel functions to find separating hyper-planes in high-dimensional spaces (Colas and Brazdil, 2006). SVM is difficult to interpret, and there have to be many parameters that need to be set for performing the classification and one parameter that performs well in one task might perform poorly in other tasks (Shah et al., 2020). Therefore, many information retrieval systems use decision trees and naive bayes. However, these models lack accuracy (Kowsari et al., 2017; Kamath et al., 2018). (2) LSTM (long-short-term memory): With the emergence of deep learning algorithms, the accuracy of text categorization has been greatly improved. Convolutional neural networks (CNN) and long short-term memory networks (LSTM) are widely used (Wang et al., 2017; Kamath et al., 2018; Luan and Lin, 2019; Yu et al., 2020). (3) The state-of-the-art pre-training language model BERT (Bidirectional Encoder Representations from Transformers): It is trained on a large network with a large amount of unlabeled data and adopts a fine-tuning approach that requires almost no specific architecture for each end task and has achieved great success in a couple of NLP tasks, such as natural language inference, and text classification (Yu et al., 2019; González-Carvajal and Garrido-Merchán, 2020; Jin et al., 2020).

### 5.4. Evaluation metric

To evaluate the performance of binary and multi-class barrier classification models, the F1-score is used as an evaluation measure.

- **F1-score:** It combines the precision and recall of a classifier into a single metric by taking their harmonic mean. It is defined as:
  $$F_{1}=\frac{2\left(Precision*Recall\right)}{Precision+Recall}$$

## 6. Results

In this section, we present the experimental results comparing simple (LR, SVM, DT, RF, kNN), deep learning (LSTM), and transformers (BERT) for the barrier classification task.

### 6.1. Comparative analysis of the ten categories

We compare the results of all ten news categories based on the evaluation metric F1-score. Since the results of LR among the five (LR, SVC, NB, DT, and kNN) classical machine learning algorithms were higher in all categories, we exclude the others. Table 3 compares the results of LR, LSTM, and BERT with our proposed approach that is based on common-sense-based semantic knowledge and sentiment. The words PM-LSTM (proposed model LSTM) and PM-BERT (proposed model BERT) mean the usage of LSTM and BERT utilizing our approach with the inference-based semantic knowledge and sentiments. For the cultural barrier, F1-scores using BERT or LSTM with common-sense-based semantic knowledge and sentiment are higher than LR, LSTM, and BERT for business, computers, games, health, home, recreation, science, shopping, society, and sports (with an improvement of 0.02, 0.05, 0.01, 0.09, 0.11, 0.14, 0.09, 0.12, 0.06, and 0.03, respectively). For the economic barrier, F1-scores are higher than LR, LSTM, and BERT for business, computers, health, home, and sports (with an improvement of 0.06, 0.03, 0.1, 0.14, and 0.01, respectively). For the political barrier, F1-scores are higher than LR, LSTM, and BERT for business, computers, games, health, home, recreation, science, shopping, and society (with an improvement of 0.12, 0.28, 0.08, 0.13, 0.21, 0.07, 0.1, 0.14, and 0.24, respectively). For the linguistic barrier, F1-scores are higher than LR, LSTM, and BERT for science, and society (with an improvement of 0.26 and 0.24, respectively). For the geographical barrier, F1-scores are higher than LR, LSTM, and BERT for business, computers, health, home, recreation, and society (with an improvement of 0.03, 0.44, 0.25, 0.17, 0.26, and 0.28, respectively).

**Table 3:** F1-score of the five different machine learning algorithms (LR, LSTM, BERT, PM-LSTM, and PM-BERT) for the ten different categories (business, computers, games, health, home, recreation, science, shopping, society, and sports).

**Model**	**Category**	**Cul**	**Eco**	**Pol**	**Ling**	**Geo**	**Category**	**Cul**	**Eco**	**Pol**	**Ling**	**Geo**
**LR**	Business	0.40	0.48	0.71	0.73	0.61	Recreation	0.37	0.30	0.59	0.73	0.57
	Computers	0.42	0.35	0.58	0.63	0.56	Science	0.42	0.42	0.62	0.71	0.65
	Games	0.52	0.35	0.59	0.59	0.60	Shopping	0.36	0.27	0.49	0.61	0.52
	Health	0.36	0.40	0.58	0.67	0.64	Society	0.40	0.45	0.62	0.68	0.62
	Home	0.39	0.57	0.49	0.68	0.59	Sports	0.44	0.28	0.59	0.62	0.57
**LSTM**	Business	0.19	0.28	0.49	0.55	0.47	Recreation	0.20	0.39	0.48	0.41	0.47
	Computers	0.20	0.08	0.49	0.52	0.46	Science	0.20	0.20	0.48	0.43	0.43
	Games	0.14	0.29	0.74	0.70	0.48	Shopping	0.23	0.44	0.49	0.44	0.49
	Health	0.18	0.17	0.49	0.59	0.53	Society	0.20	0.27	0.49	0.46	0.48
	Home	0.19	0.21	0.49	0.59	0.63	Sports	0.26	0.43	0.48	0.43	0.49
**BERT**	Business	0.42	0.54	0.62	0.74	0.50	Recreation	0.32	0.29	0.74	0.81	0.59
	Computers	0.38	0.30	0.47	0.75	0.66	Science	0.39	0.47	0.68	0.78	0.60
	Games	0.40	0.65	0.77	0.85	0.68	Shopping	0.34	0.44	0.49	0.63	0.50
	Health	0.38	0.54	0.66	0.79	0.67	Society	0.39	0.51	0.49	0.73	0.48
	Home	0.32	0.60	0.54	0.81	0.67	Sports	0.48	0.50	0.48	0.76	0.66
**PM-LSTM**	Business	0.19	0.28	0.49	0.43	0.48	Recreation	0.21	0.47	0.48	0.44	0.46
	Computers	0.21	0.06	0.49	0.46	0.47	Science	0.19	0.21	0.48	0.74	0.43
	Games	0.18	0.22	0.48	0.40	0.48	Shopping	0.18	0.50	0.49	0.50	0.48
	Health	0.20	0.27	0.48	0.43	0.46	Society	0.19	0.25	0.49	0.64	0.48
	Home	0.20	0.27	0.49	0.45	0.48	Sports	0.27	0.22	0.48	0.44	0.48
**PM-BERT**	Business	0.48	0.66	**0.97**	0.80	0.96	Recreation	**0.66**	0.35	0.74	0.48	0.65
	Computers	0.46	0.28	0.54	**0.97**	0.70	Science	0.47	0.53	**0.97**	0.78	0.66
	Games	0.33	**0.72**	0.48	0.47	0.72	Shopping	0.45	0.50	**0.97**	0.67	0.56
	Health	0.46	0.60	0.72	0.82	**0.97**	Society	0.52	0.69	0.55	0.73	**0.97**
	Home	0.41	0.66	0.54	0.83	0.73	Sports	0.49	0.50	0.54	0.79	0.63

The bold values indicate the highest F1-scores that have been achieved by a method for a category.

The results presented in Table 3 indicate that the best results for each barrier are obtained by PM-BERT and BERT, closely followed by PM-LSTM. The LR performs a little less compared to the other algorithms tested. Moreover, it can be seen that the obtained F1-scores vary significantly across different categories. While the obtained F1-score is very high for the two categories (health and society) of the geographical barrier and for the three categories (business, shopping, and science) of the political barrier, and a quite good score is obtained for the recreation category of the cultural barrier, the games category of the economic barrier, and the computers category of the linguistic barrier, the score is low comparatively for the other categories of the different barriers.

The best results obtained for the task of classifying the barriers for the ten different categories are a direct consequence of the class distribution and sentiments of the classes, to some extent. As far as the results are concerned for all the barriers, we see that the highest F1-score is produced for the health (0.97) and society (0.97) categories of the geographical barrier, recreation (0.66) category of the cultural barrier, games (0.72) category of the economic barrier, computers (0.97) category of the linguistic barrier, and business (0.97), shopping (0.97), and science (0.97) categories of the political barrier. The F1-score for the society category of the geographic barrier and business category of the political barrier is as high as 0.97. An obvious reason for this is the fact that the data is heavily imbalanced, with 95 and 91% instances of majority classes. However, both are showing improvements. This can be due to a slight variation in sentiments across its binary classes. For the health category of the geographical barrier, and the shopping and science category of the political barrier, the class distribution is not very imbalanced (78, 85, and 75% instances of the majority class), but the F1-score is really high, which means PM-BERT is best suited for these categories. Regarding its best results, it might be possible that sentiments across these binary classes have variations, such as the label “Crossed-GB” having more positive and fewer negative instances than “Not-crossed-GB” and vice versa. Similarly, the shopping and science categories of the political barrier consist of more news headlines with negative sentiments for the label “Crossed-PB” and vice versa for the label “Not-crossed-PB.” PM-BERT has proved to be best suited for the classification of computers and games categories of the linguistic and the economic barriers. We see that the data is quite balanced for computers category of the linguistic (75 and 25% instances of “Crossed-LB,” “Not-crossed-LB” respectively) and games category of the economic barrier (29, 49, and 22% instances of “information-crossing,” “information-not-crossing,” and “unsure” classes, respectively). Looking into the sentiments of each class of computers category (see Figures 2, 3), we observe that one has more news headlines with positive headlines and less negative news headlines and vice versa. However, for the games category of the economic barrier, sentiments are varying across all three classes: 12, 25, and 50% news headlines with positive, neutral, and negative sentiments, respectively, for the label “Information-crossing.” The label “Unsure” does not have news headlines with neutral sentiments, whereas all the news headlines labeled “Information-not-crossing” have only positive sentiments. For the recreation category of the cultural barrier, although the distribution of positive, neutral, and negative sentiments across all three barriers is almost equal and the class distribution is balanced (10, 44, and 46% instances for “Information-crossing,” “Information-not-crossing,” and “Unsure,” respectively, the PM-BERT performs really well (0.66 F1-score).

### 6.2. Comparative analysis of three types of algorithms

**Figure 15:**
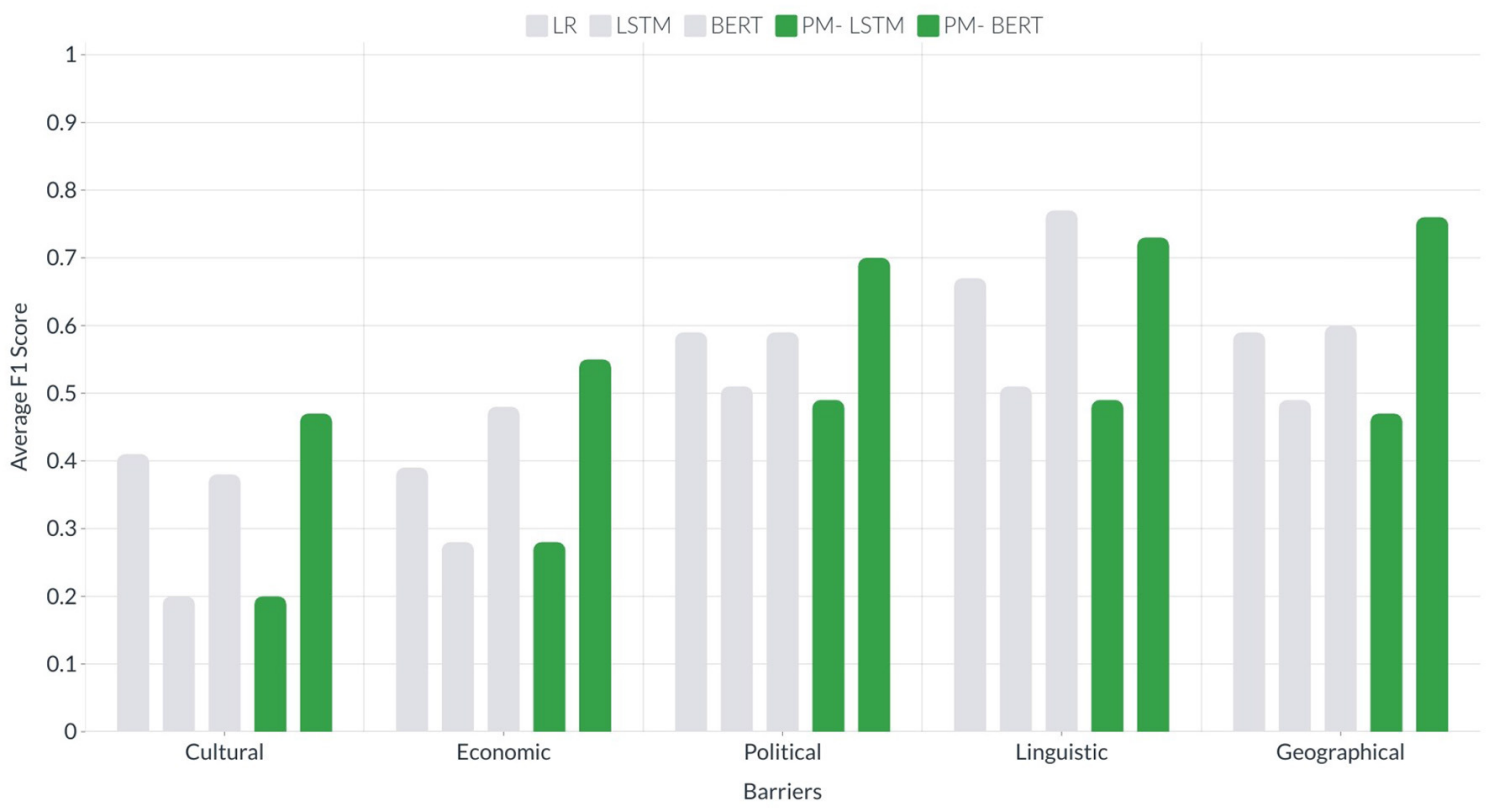
It presents bars of two colors for each barrier. The green bars show the average F1-scores of all the ten categories for LSTM and BERT using common-sense-based semantic knowledge and sentiments. The gray bars show the average F1-scores of all the ten categories for LR, LSTM, and BERT using only headline text. The x-axis shows the groups of bars for all five barriers, whereas the y-axis shows the average F1-score.

After discussing the results of all ten news categories, we compare all five different types of barriers using the average F1-scores of all ten categories. Figure 15 presents the comparison of the average F1-score of all the categories. The highest average F1-score for the cultural barrier is obtained using PM-BERT (0.47) and BERT (0.38), whereas this score was low using PM-LSTM (0.22). For the economic, political, and geographical barriers, the average F1-score obtained using PM-BERT (0.55, 0.70, and 0.76 respectively) was followed by a slight lower average F1-score using BERT (0.48, 0.59, and 0.60, respectively) and then PM-LSTM (0.28, 0.49, and 0.57, respectively).

With regards to the linguistic barrier, the highest average F1-score was achieved using BERT instead of PM-BERT or PM-LSTM, which is quite interesting and questionable. Instead of average, we look for the F1-score of all the ten categories of this barrier. The obtained F1-score for the eight categories (business, computers, health, home, science, shopping, sports, and society) using PM-BERT is higher then that using BERT (using PM-BERT : 0.80, 0.97, 0.82, 0.83, 0.78, 0.67, 0.73, and 0.79, respectively; using BERT : 0.74, 0.75, 0.79, 0.81, 0.78, 0.63, 0.73, and 0.76, respectively). However, the F1-score for the game and recreation categories of the linguistic barrier using PM-BERT (0.47 and 0.48, respectively) was considerably lower than BERT (0.85 and 0.81, respectively). On the other hand, the obtained average F1-score is better for four barriers, including economic, political, linguistic, and geographical, than the cultural barrier, which is just under 0.5. However, for the other barriers, it is well over 0.50 and extends to near 0.80. To answer the **Q3**, we can say that our proposed methods (LSTM and BERT with semantic knowledge) outperform for the four cultural, economic, political, and geographical barriers.

## 7. Analysis and discussion

Experiments with the novel approach on the ten different kinds of news and the five different barriers have brought some insights regarding information spreading. In order to support the hypothesis, we have set three research questions.

To answer the first research question (Do the sentiments of the headlines of different topics vary across the different barriers?), We compare the sentiments of the headlines for all the categories across the five barriers, performing sentiment analysis at the granularity of ten negative and ten positive points as well as at overall negative, positive, and neutral sentiment (see Figures 2–5). The comparative analysis indicates that the political barrier has been crossed by the news with positive sentiments whereas for the other four barriers (linguistic, geographical, cultural, and economic), news with positive sentiments is not crossing the barriers. With regard to the binary class classification for the political, linguistic, and geographical barrier, we see that the news headlines that are labeled as crossing the barrier have more instances of negative sentiments, whereas the news headlines that are labeled as not crossing the barrier have more instances of positive sentiments. With regard to the ternary class classification for the economic and cultural barriers, the sentiments were the same as in the binary class classification, where the news headlines are labeled as crossing or not crossing the barriers. However, for the news headlines that are labeled as unsure, there were negative and positive sentiments about the economic and cultural barriers, respectively. The implication for the general audience is that the news with different categories cross the barriers. But the news that cross the political barrier are mostly of positive sentiments. Moreover, another interesting method that can be helpful to support this classification is to have domain-specific phrases. For instance, in case of economic barrier and science category, a pre-defined phrases along with their semantic description can be detected in the headlines. These phrases can further support this classification of either what type of science-related news are crossing the economic barrier or vice verse. One of the available datasets includes *Fintech key-phrase dataset* (Jin et al., 2023a).

To answer the second research question (What are the properties (statistics and ratio) of the common-sense knowledge relations in news headlines to different topics?), we find the intersection between the inferences belonging to different barriers and categories (see Figures 6–8). The results suggest that although inferences are being shared among the classes, there are some unique inferences for each class. Similarly, the same fact exists between the different categories. Therefore, it might be possible that it will help to improve the classification results. The results of the annotation show that there are variations in class distributions across different categories. Therefore, we use stratified sampling to maintain the class proportion in the train and test sets (see Figures 13, 14).

To answer our third research question (Which classification methods (classical or deep learning methods) yield the best performance to the barrier classification task?), We perform classification with classical machine learning methods, including Logistic Regression (LR), Naive Bayes (NB), Support Vector Classifier (SVC), k-nearest Neighbor (kNN), and Decision Tree (DT). Afterward, we perform classification with and without inferences using LSTM and BERT. We evaluate the models using the F1-score (see Section 5.4). We analyzed the classification results by comparing the ten categories (see Section 6.1) and three types of classification methods (see Section 6.2).

The results suggest that our proposed methods (LSTM and BERT with inferences based semantic knowledge and sentiments) offer better performance for the four barriers (cultural, economic, political and geographical).

## 8. Conclusion and perspective

In this paper, we focused on the classification of barriers to the spreading of news by utilizing semantic knowledge in the form of common-sense knowledge and sentiments. We consider the news related to the ten different categories (business, computers, games, health, home, recreation, science, shopping, society, and sports). After completing the automatic annotation of news data for the five barriers, including cultural, economic, political, linguistic, and geographical (binary class classification of the linguistic, political, and geographical barrier and ternary class classification of the cultural and political barrier), we perform classification with classical machine learning methods (LR, NB, SVC, kNN, and DT), deep learning (LSTM) and transformer-based methods (BERT). Our findings suggest that common-sense based semantic knowledge and sentiments help in achieving a higher F1-score. The classification of news across the barrier can help to recommend the news belonging to different categories and to identify the trends of different kinds of news across different barriers. The main theoretical contributions of this work are an approach to information barrier annotation based on news meta-data and labeling and classifying the news, including semantic knowledge across different barriers (cultural, economic, political, linguistic, and geographical). The annotation process includes meta-data extraction that requires too many requests to find the corresponding Wikipedia URLs for news publishers. Although many news publishers, including some local news publishers, do not have an entry in the Wikipedia database, popular and a significantly large number of news publishers do have their profiles available at the Wikipedia-Infobox. The annotation process and data statistics demonstrate that this approach to extracting profiles of news publishers is feasible to perform barrier classification to news spreading as well as for other important tasks such as understanding fake news propagation. The labeling process involves the demographic values and profile of news publishers, such as cultural and economic differences, political alignment, and publishing language. To the best of our knowledge, our proposed approach is the first of its kind to the classification of barriers to the spreading of news. There are basically two practical contributions: (1) an annotated data set, and (2) an approach to the classification of barriers to the spreading of news based on semantic knowledge, including a wide range of common sense knowledge and sentiments of news headlines. Since the existing work lacks an annotated dataset for this task, it presents an annotated data set for the classification of barriers to the spreading of news. It presents the sentiment analysis of annotated news headlines as well as the properties of common-sense knowledge relations in news headlines. Our experimental evaluation shows that deep learning (LSTM) and transformer-based methods (BERT) can be useful for classifying barriers using common-sense-based knowledge and sentiments.

In the future, we plan to analyze the performance of prompt learning and GPT-based generative classification models for barrier classification to the spreading of news. Moreover, currently, geographical barrier is calculated in a binary way. In the future, we would like to extend the classes based on the distance between countries and time zone. Similarly, for the political and linguistic barriers, we will incorporate more information while annotating the news.

## Data availability statement

The datasets generated for this study can be found in the GitHub repository via the following link: https://github.com/abdulsittar/BC-Inferences-Sentiments.git.

## Author contributions

AS: methodology, data curation, writing—original draft preparation, software, and writing—reviewing and editing. DM: supervision, validation, and writing—reviewing and editing. MG: conceptualization and funding acquisition. All authors contributed to the article and approved the submitted version.

## Footnotes

^1^ https://brightdata.com/

^2^ https://github.com/abdulsittar/BC-Inferences-Sentiments.git

^3^ https://github.com/EventRegistry/event-registry-python/blob/master/eventregistry/examples/QueryArticlesExamples.py

^4^ https://dmoz-odp.org/

^5^ https://eventregistry.org/documentation?tab=terminology

^6^ https://www.prosperity.com/
